# Comparison of *Arachis monticola* with Diploid and Cultivated Tetraploid Genomes Reveals Asymmetric Subgenome Evolution and Improvement of Peanut

**DOI:** 10.1002/advs.201901672

**Published:** 2019-11-28

**Authors:** Dongmei Yin, Changmian Ji, Qingxin Song, Wanke Zhang, Xingguo Zhang, Kunkun Zhao, Charles Y. Chen, Chuantang Wang, Guohao He, Zhe Liang, Xingli Ma, Zhongfeng Li, Yueyi Tang, Yuejun Wang, Ke Li, Longlong Ning, Hui Zhang, Kai Zhao, Xuming Li, Haiyan Yu, Yan Lei, Mingcheng Wang, Liming Ma, Hongkun Zheng, Yijing Zhang, Jinsong Zhang, Wei Hu, Z. Jeffrey Chen

**Affiliations:** ^1^ College of Agronomy Henan Agricultural University Zhengzhou 450002 China; ^2^ Biomarker Technologies Corporation Beijing 101300 China; ^3^ Hainan Key Laboratory for Biosafety Monitoring and Molecular Breeding in Off‐Season Reproduction Regions Institute of Tropical Bioscience and Biotechnology Chinese Academy of Tropical Agricultural Sciences Haikou 571101 China; ^4^ State Key Laboratory of Crop Genetics and Germplasm Enhancement Nanjing Agricultural University Nanjing 210095 China; ^5^ Department of Molecular Biosciences and Center for Computational Biology and Bioinformatics The University of Texas at Austin Austin 78705 USA; ^6^ State Key Lab of Plant Genomics Institute of Genetics and Developmental Biology INASEED Chinese Academy of Sciences Beijing 100101 China; ^7^ College of Agriculture Auburn University Auburn AL 36849 USA; ^8^ Shandong Peanut Research Institute Qingdao 266000 China; ^9^ Department of Agricultural and Environmental Sciences Tuskegee University Tuskegee AL 36088 USA; ^10^ Centre for Organismal Studies University of Heidelberg D‐69120 Heidelberg Germany; ^11^ National Key Laboratory of Plant Molecular Genetics Center for Excellence in Molecular Plant Sciences Institute of Plant Physiology and Ecology Shanghai Institutes for Biological Sciences Chinese Academy of Sciences Shanghai 200032 China

**Keywords:** domestication, evolution, peanut, polyploidy, sequence

## Abstract

Like many important crops, peanut is a polyploid that underwent polyploidization, evolution, and domestication. The wild allotetraploid peanut species *Arachis monticola* (*A. monticola*) is an important and unique link from the wild diploid species to cultivated tetraploid species in the *Arachis* lineage. However, little is known about *A. monticola* and its role in the evolution and domestication of this important crop. A fully annotated sequence of ≈2.6 Gb *A. monticola* genome and comparative genomics of the *Arachis* species is reported. Genomic reconstruction of 17 wild diploids from AA, BB, EE, KK, and CC groups and 30 tetraploids demonstrates a monophyletic origin of A and B subgenomes in allotetraploid peanuts. The wild and cultivated tetraploids undergo asymmetric subgenome evolution, including homoeologous exchanges, homoeolog expression bias, and structural variation (SV), leading to subgenome functional divergence during peanut domestication. Significantly, SV‐associated homoeologs tend to show expression bias and correlation with pod size increase from diploids to wild and cultivated tetraploids. Moreover, genomic analysis of disease resistance genes shows the unique alleles present in the wild peanut can be introduced into breeding programs to improve some resistance traits in the cultivated peanuts. These genomic resources are valuable for studying polyploid genome evolution, domestication, and improvement of peanut production and resistance.

## Graphical abstract

The wild allotetraploid peanut species *Arachis monticola* (*A. monticola*) is an important and unique link from the wild diploid species to cultivated tetraploid species in the *Arachis* lineage. A fully annotated sequence of the *A. monticola* genome and comparative genomics of *Arachis* species are presented, which reveals asymmetric subgenome evolution and genome‐assisted improvement of peanut production and resistance.

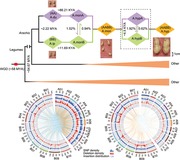

## 1 Introduction

Peanut (*Arachis hypogaea* L.) is a major oilseed legume crop of global importance, and over 95% of cultivated areas are in Asia and Africa. Approximately 42 million tons of peanuts are produced and consumed annually by all human societies across the world. Originating from South America, the genus *Arachis*, including ≈80 species, has been classified into nine sections and shows a unique reproductive trait of subterranean fruits.^1^ Section *Arachis* is genetically diverse and consists of 30 diploid species and two tetraploids, one wild (*Arachis monticola* (*A. monticola*)) and the other cultivated (*Arachis hypogaea* (*A. hypogaea*)).^2^ Hybridization between two diploid species like *Arachis duranensis* (*A. duranensis*) (AA, 2*n* = 2*x* = 20) and *Arachis ipaensis* (*A. ipaensis*) (BB, 2*n* = 2*x* = 20) gave rise to a wild tetraploid species, *A. monticola* (AABB, 2*n* = 4*x* = 40), which was domesticated into the cultivated tetraploid crop.^3^, ^4^, ^5^, ^6^, ^7^, ^8^ Thus, *A. monticola* is an important link from the wild diploid species to cultivated tetraploid species in the *Arachis* lineage.

Polyploidy is a widespread evolutionary process and has played a key role in plant speciation and domestication.^9^, ^10^, ^11^ As a result, many crop plants including wheat, cotton, and canola are polyploids, and their genomes have been sequenced.^12^, ^13^, ^14^ Peanuts represent an important genetic model for understanding polyploid genome evolution and crop domestication, as wild diploid ancestors, wild and cultivated tetraploid species are available.^2^, ^15^, ^16^, ^17^, ^18^, ^19^ *A. ipaensis* and *A. duranensis* shared over 80% the synteny regions with the major rearrangements occurring in the A‐genome lineage. *A. duranensis* is nearly identical to the B subgenome of *A. hypogaea*, which has experienced a genetic bottleneck and reproductive isolation.^15^ However, the mode of polyploid evolution and the mechanism for subgenome evolution and trait domestication in tetraploid peanuts remain unknown.

Polyploids often exhibit more genome structural variation than their diploid progenitors.^20^ Structural variants (SVs) represent large rearrangements of genomic types including deletions, insertions, inversions, duplications, and copy number variations.^21^ Some studies have shown the effect of SVs on gene expression and phenotypic traits.^22^, ^23^, ^24^ This type of variation has not been characterized in evolution and domestication of peanuts from wild diploids to wild and cultivated tetraploids.

In this work, we fully annotated a high‐quality sequence of the wild tetraploid peanut genome and performed comprehensive analyses of genomes and gene expression from diploid ancestors to wild and cultivated tetraploids. We investigated subgenome orgin, evolution, structural variation, functional divergence, pod domestication, and disease resistance in peanuts. Together, these genomic resources should provide new tools for accelerating the genomic improvement of peanuts, which will enhance global oil and food security to feed a growing population in the world.

## 2 Results

### 2.1 Comparative Analyses of the *A. Monticola* Genome and Cultivated Tetraploids

Previously, we have assembled the whole genome sequence of wild peanut *A. monticola*, a tetraploid species, based on a combined set of data using illumina short read sequencing, single molecule real time (SMRT) sequencing, Bionano genome map, and high throughput chromosome conformation capture (Hi‐C) technologies.^2^ In this study, we further performed full annotation of the assembled genome. The *A. monticola* genome has ≈73.2% of transposable elements (TEs) including the most abundant LTR/Gypsy (45.4%) and PLE|LARD (19.9%) elements (Table S1, Supporting Information), which was slightly higher than those in the cultivated peanut (64%) and diploid progenitors *A. duranensis* (61.7%) and *A. ipaensis* (68.5%).^15^ The genome has 11 569 pseudogenes with frame shift and/or premature stop codon (Table S2, Supporting Information). We also identified 15 431 noncoding RNAs (ncRNAs), including 1202 tRNAs, 485 rRNAs, 116 miRNAs, 110 snRNAs, and 13 500 siRNAs, occupying 1.91 Mb of *A. monticola* genome (Table S3, Supporting Information). Using a comprehensive strategy of evidence‐based and ab initio gene predictions, we identified 74 907 gene models in *A. monticola* with more than 91% BUSCO completeness, including 34 117 in the A subgenome and 38 566 in the B subgenome (Table S4a,b, Supporting Information). The majority (30 306 in A subgenome and 34 591 in B genome) of them were supported by the homology to known proteins and/or existence of known functional domains (Table S4c, Supporting Information). The genomic landscape of TEs, pseudogenes, protein coding genes, ncRNAs, expression patterns, and sequence variation was visualized with Circos. The distribution of TEs and pseudogenes was similar in the two subgenomes, forming dense accumulation in pericentromeric regions (Figure S1, Supporting Information). Genome‐wide pooled transcriptome data showed high active transcription near chromosome ends, consistent with the gene density distribution (Figure S1, Supporting Information).

The availability of reference genomes from both wild and cultivated tetraploids enabled us to explore genomic differences between subgenomes in the tetraploids and their respective A and B‐genome‐like diploids.^2^, ^15^, ^16^, ^17^, ^18^, ^19^ The *A. monticola* genome showed higher levels of collinearity in both euchromatic and pericentromeric regions of homoeologous chromosomes than A and B‐genome‐like diploids (Figure S1, Supporting Information).^15^ Between subgenomes, the B subgenome has higher levels of co‐linearity with the diploid B genome than the A subgenome with its diploid A genome (Figures S2 and S3, Supporting Information). A few large rearrangements have been identified in A07, A08, B07, and B08 of *A. monticola*, as previously reported in diploid genomes (Figure S1, Supporting Information).^15^

The direct genome comparison analysis allowed a genomic characterization of structural variations of inversions and translocations. Compared with the sequences of diploid ancestors, we observed many inversions and translocations in wild *A. monticola* genome (Figures S2a and S3a, Supporting Information), suggesting the rapidly genome evolution of the relatively young tetraploid species. Notably, genomic organization including these structural variations is highly maintained from the wild *A. monticola* to cultivated *A. hypogaea* tetraploid species and is more conservative than that comparing to their progenitor‐like diploids (Figures S2a and S3a, Supporting Information), supporting origin of the domesticated peanut from *A. monticola*.^3^, ^4^, ^5^, ^6^, ^7^, ^8^ Besides, there are several large inversions in A03, A07, A09, B05, and B10 of *A. monticola*, which are not observed in *A. hypogaea*, suggesting the possible introgression events occurred from wild diploids to cultivated *A. hypogaea* (Figures S2a and S3a, Supporting Information). These large inversions are present with discrete chromatin interactions around breakpoints by mapping Hi‐C links between species (Figures S2b and S3b, Supporting Information), and similar Hi‐C interaction maps have been used to identify large‐scale chromosomal rearrangements in tetraploid cotton.^25^ The differences between the A and B subgenomes of the wild and cultivated peanuts and their respective diploids may lead to the debate about the origin of tetraploid peanuts, which is more likely resolved in this study.^26^, ^27^, ^28^, ^29^

Overall transcript levels are similar between subgenomes (Figure S4a, Supporting Information). Sequence divergence of orthologous gene pairs between A subgenome and *A. duranensis* (median = 5.67) is significantly higher than that between B subgenome and *A. ipaensis* (median = 2.51) (Wilcoxon rank‐sum test, *P* < 0.01) (Figure S4b, Supporting Information), consistent with data in cultivated lines.^15^ In *A. monticola*, A subgenome has slightly higher nonsynonymous (*Ka*) and synonymous (*Ks*) mutation rates than B subgenome (Figure S5, Supporting Information), while the heterozygosity rate is lower in the A subgenome than in the B subgenome of wild and cultivated tetraploid peanuts (Figure S4c, Supporting Information).

### 2.2 Monophyletic Origin and Diversification of A and B Subgenomes

**Figure 1:**
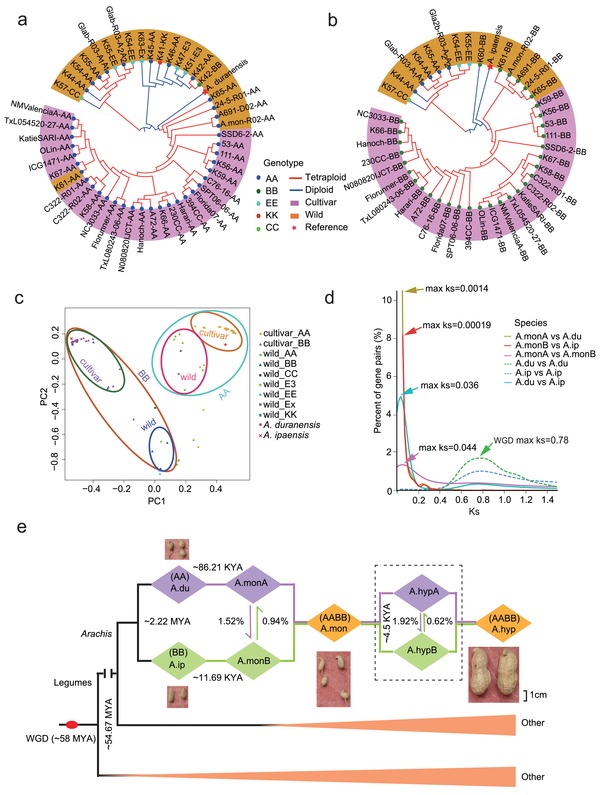
Subgenome origins and phylogenetic analysis of wild and cultivated peanut lines. a) Phylogenetic tree showing monophyletic relationship of A subgenomes in allotetraploid peanuts. b) Phylogenetic tree showing monophyletic relationship of B subgenomes in allotetraploid peanuts. c) Principle component analysis (PCA) of 47 resequenced accessions. Tetraploid accessions were separated into two groups with their respective progenitors. d) Distribution of *Ks* values for orthologous gene sets among diploid progenitors (*A. duranensis*: A.du; and *A. ipaensis*: A.ip) and tetraploids (*A. monticola*: A.mon; and *A. hypogaea*: A.hyp). e) Reconstruction of an evolutionary model for wild and cultivated peanuts. Ancestral whole genome duplication (WGD) event of legumes is shown (red node).

We resequenced genomes of 17 wild diploids from AA, BB, EE, KK, and CC groups and 30 wild and cultivated tetraploids, and performed phylogenetic analyses (Table S5, Supporting Information). All diploid accessions were placed in two separate (wild and cultivated) groups (**Figure** **1**a,b). This classification is also supported by principal component analysis (PCA) (Figure 1c). The cultivated tetraploids were closest to wild tetraploids, suggesting domestication of *A. hypogaea* from *A. monticola*. A and B subgenomes of both tetraploids were rooted with the A‐genome‐like diploid *A. duranensis* and the B‐genome‐like diploid *A. ipaensis*, respectively (Figure 1a–c), indicating a monophyletic origin of A and B subgenomes.

During evolution of legumes, an ancestral whole genome duplication (WGD) occurred at ≈58 million years ago (MYA) (*Ks* = 0.78 in *Archis*). The peak *Ks* (0.044) between *A. monticola* subgenomes is slightly higher than that between two ancestors (0.036) (Figure 1d). A rate of synonymous changes in tetraploid peanut is ≈1.2 times faster than that of its diploid ancestors (Figure 1d), indicating that the formation of tetraploids accelerated the sequence divergence between two subgenomes. Using available sequence data, we reconstructed a model for allotetraploid peanut evolution (Figure 1e). *A. duranensis* and *A. ipaensis* diverged from *Arachis* lineage at ≈2.2 MYA, as previously estimated.^15^ The wild peanut (*A. monticola*) was formed ≈11 690 years ago by hybridization between *A. duranensis* and *A. ipaensis*, followed by chromosome doubling. Domestication of *A. hypogaea* took place ≈4500 years ago, and the modern cultivated tetraploid has many improved agronomic traits, especially in pod size, but loses some resistance traits deposited in wild relatives.

### 2.3 Asymmetric Subgenome Evolution and Expression Divergence in Tetraploid Peanuts

**Figure 2:**
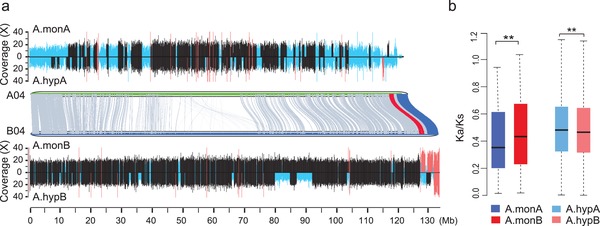
Asymmetric subgenome evolution of allotetraploid peanuts. a) Homoeologous sequence exchanges (HSEs) between chromosome A04 and B04. Segmental HSEs were revealed based on sequence read coverage from wild and cultivated allotetraploid peanuts to *A. duranensis* and *A. ipaensis* genomes, respectively. A duplication of genomic segments (red) is represented by a greater coverage for a given segment than the rest of the genome (black) or a deletion (blue) with less or no coverage. b) Selection bias between A and B subgenomes of wild and cultivated peanuts. The estimates are based on *Ka*/*Ks* values (Wilcoxon rank‐sum test and permutation test with 10 000 permutations, **P < 0.01).

The domestication timeframe of *A. hypogaea* is similar to that of rapeseed (*Brassica napus* (*B. napus*)), which is accompanied by abundant homoeologous sequence exchanges (HSEs).^13^ In peanuts, total HSE ratios between subgenomes of *A. monticola* and that of *A. hypogaea* were 2.46% and 2.54%, respectively (Figure 1e; and Table S6, Supporting Information). HSEs from A to B subgenomes were higher than those from B to A subgenomes in both *A. monticola* and *A. hypogaea* (**Figure** **2**a), suggesting asymmetric HSEs contributing to the diversification of *A. monticola* and *A. hypogaea*. The HSEs from A to B subgenomes in *A. monticola* were enriched with the genes in flavonoid biosynthesis and circadian rhythm pathways (hypergeometric test, *P* < 0.01) (Table S7, Supporting Information), suggesting a role for asymmetric HSEs in biological function.

Although the divergence time (≈2.16 MYA) was similar between *A. duranensis* and *A. ipaensis*, as previously estimated,^15^ A subgenome of *A. monticola* (1113) showed 1.5‐fold more contracted gene families than the wild *A. duranensis* (721), whereas B subgenome of *A. monticola* (1034) had 1.5‐fold more expanded gene families than the wild *A. ipaensis* (703) (Figure S6 and Table S8, Supporting Information). In *A. monticola*, gene families in starch and sucrose metabolism, linoleic acid metabolism, and cutin, suberin and wax biosynthesis pathways were contracted in the A subgenome but expanded in B subgenome; conversely, glucosinolate biosynthesis pathways were expanded in the A subgenome but contracted in the B subgenome (Table S8, Supporting Information). This indicates asymmetric gene family expansion and contraction between subgenomes after polyploidization.

To distinguish homoeologous expression diversity, we identified 20 516 pairs of homoeologs between A and B subgenomes of *A. monticola* and investigated their expression variation. No overall expression bias was observed between A and B genomes of *A. monticola* in pooled transcriptome data (Figure S4a, Supporting Information), which is consistent with the results from other polyploid crops, such as cotton (*Gossypium hirsutum*),^12^ rapeseed (*B. napus*),^13^ senvy (*Brassica juncea* (*B. juncea*)),^30^ and wheat (*Triticum aestivum*).^31^ However, homoeolog expression bias was observed during pod development. Comparing transcriptome data of wild *A. monticola* (small pod) with cultivated *A. hypogaea* Hua8106 (median pod) and Hua8107 (large pod), we found that 5571, 5542, and 5654 homoeologous gene pairs (≈27%) displayed expression bias (Tables S9–S12, Supporting Information). Interestingly, expression bias occurred in 11 homoeologous gene pairs within flavonoid biosynthesis pathway, all of which were associated with the B subgenome in *A. monticola* and *A. hypogaea* during pod development, indicating homoeologous expression divergence in response to pod selection (Figure S7, Supporting Information).

Consistent with the expression divergence, the selection acts differently on two subgenomes between *A. monticola* and *A. hypogaea*. The *Ka*/*Ks* values of homoeolog pairs were significantly lower in the A (median = 0.357) than in the B (0.441) subgenomes of *A. monticola* (Wilcoxon rank‐sum test, *P* < 2.2 × 10^−16^ and permutation test with 10 000 permutations, *P* < 2.2 × 10^−16^), whereas *Ka*/*Ks* values were significantly higher in the A (0.481) than in the B (0.465) subgenomes of *A. hypogaea* (Wilcoxon rank‐sum test, *P* < 1.1 × 10^–4^ and permutation test with 10 000 permutations, *P* < 0.013) (Figure 2b; and Figure S8, Supporting Information). This suggests that natural selection may be biased toward the B subgenome in wild *A. monticola*, but domestication has larger effects on the A subgenome of cultivated *A. hypogaea*. These data suggest a role for asymmetric selection in expression divergence between homoeologous genes and subgenomes of peanuts.^32^, ^33^, ^34^, ^35^

### 2.4 Structural Variation between Tetraploid Subgenomes during Domestication

**Figure 3:**
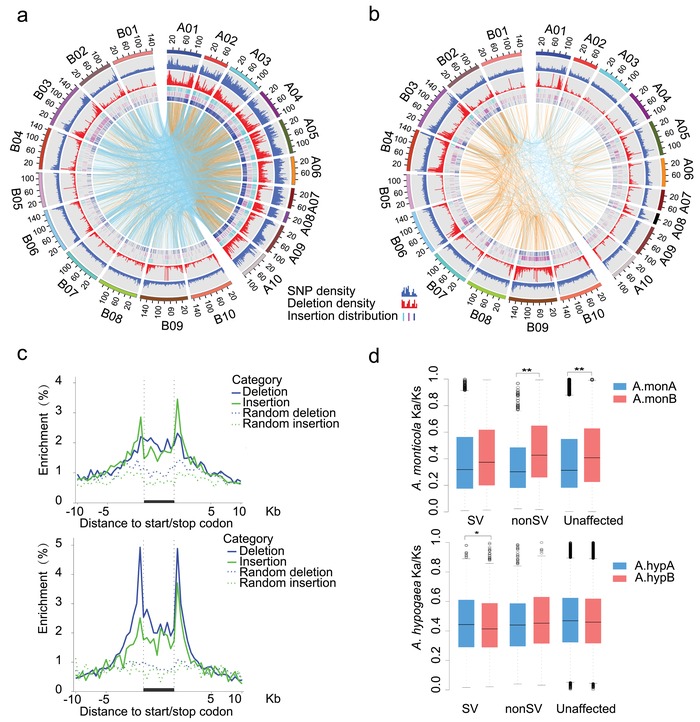
Asymmetric SV accumulation from wild diploids to allotetraploid peanuts. a) SV distribution of wild allotetraploid peanuts. The tracks (from outside to inside) indicate chromosomes, SNP density, deletion density, and insertion distribution (purple: tandem duplication insertion; blue: inter or intra‐insertion; and cyan: other insertion). Lines in the inner track show the connections between the insertion and its corresponding locus. The orange and cyan lines represent intra and inter‐subgenome insertions, respectively. b) SV distribution of cultivated allotetraploid peanuts as in (a). c) SV abundance in potential regulatory regions of protein coding genes (solid lines) and randomly selected genes in the genome (dashed lines). Only deletions (blue) and insertions (green) are shown for wild (upper panel) and cultivated (lower panel) peanuts. d) Selection bias among homoeologous genes in SV, non‐SV, and unaffected groups (Wilcoxon rank‐sum test, **P < 0.01).

SVs including deletions and insertions could affect gene expression and phenotypic traits.^36^, ^37^ In *A. monticola*, a total of 7 753 594 single nucleotide polymorphisms (SNPs), 17 226 deletions, and 7504 insertions were identified relative to *A. duranensis* and *A. ipaensis* genomes (Table S13a, Supporting Information). The cultivated tetraploid *A. hypogaea* has fewer numbers of SNPs (3 802 245), deletions (9464), and insertions (2708) than *A. monticola* (Table S13b, Supporting Information). Interestingly, higher SNP, deletion, and insertion frequencies in the A subgenome than in the B subgenome were observed in the wild tetraploid (**Figure** **3**a), but these frequencies were lower in the A subgenome than in the B subgenome in the cultivated tetraploid (Figure 3b). The A subgenome had more intra‐subgenomic insertions than the B subgenome in wild tetraploid (Figure 3a; and Table S13c, Supporting Information), while the B subgenome had more intra‐subgenomic insertions in the cultivated tetraploid (Figure 3b; and Figures S9–S11 and Table S13c, Supporting Information). Notably, *A. hypogaea* showed more enrichment of deletions and insertions in the upstream regions of the coding sequences than *A. monticola* (Figure 3c). These results suggest the different patterns of SVs accumulation in subgenomes from wild diploids to allotetraploid peanuts.

We identified 3975 and 1838 homoeologous pairs with SVs in the wild and cultivated peanuts from 20 516 homoeologous pairs, respectively. For each pair of homoeologs between A and B‐subgenomes, we defined one gene with SVs in its upstream or gene body regions as structural variation (SV) gene and the other gene without SVs in its upstream or gene body regions as non‐SV gene. Both of genes in a homoeologous pair without SVs in their upstream or gene body were defined as unaffected genes. The distribution of *Ka*/*Ks* values among SV, non‐SV, and unaffected genes is shown in Figure 3d. In *A. monticola*, the median *Ka*/*Ks* values of SV, non‐SV, and unaffected gene sets in A subgenome were significantly lower than those in B subgenome, suggesting strong natural selection on the homoeologous genes of B subgenome. In *A. hypogaea*, SV genes in A subgenome showed higher *Ka*/*Ks* value than in B subgenome, suggesting human selection on homoeologous SV genes of A subgenome. These results revealed different impact of selection on SV genes between subgenomes during peanut domestication.

### 2.5 SVs Affect Expression of the Genes Involved in Pod Development and Domestication

To investigate the influence of SVs on pod development, we examined the expression levels of homoeologous genes of SV_nonSV and unaffected gene sets in different stages of pod development in *A. monticola* and *A. hypogaea* (Figure S12, Supporting Information). SV and non‐SV genes in the B subgenome of *A. monticola* and in A and B subgenomes of *A. hypogaea* showed more expression fold‐changes between homoeologous gene pairs than unaffected genes during all the stages of pod development. Further, we analyzed the effects of different types of SVs on homoeologous gene expression (Figure S13, Supporting Information). The observation showed that deletion had more significant effects on homoeologous gene expression changes in the B subgenome of *A. monticola* and in A and B subgenomes of *A. hypogaea* relative to insertion during pod development. These results indicate a possible role for SVs in gene expression changes during pod development.

**Figure 4:**
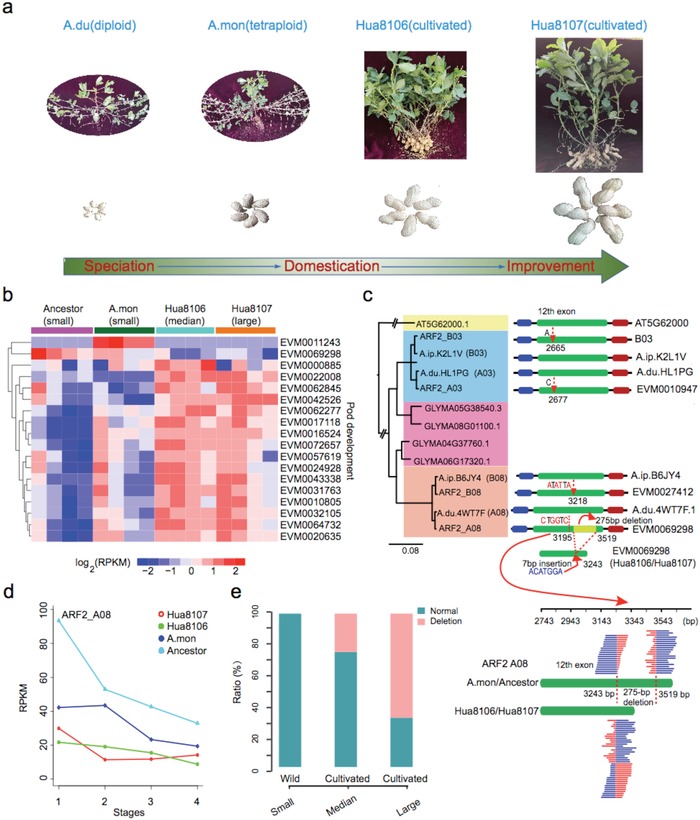
SV‐associated genes in pod development and domestication. a) Pod traits domestication from wild to cultivated lines. b) Expression profile of pod development related genes that had SVs in upstream/exon regions (UERs). c) Sequence and phylogenetic analysis of *ARF2* copies in *Arachis*, showing a 275 bp deletion that can be found in sequence reads of Hua8106 and Hua8107. d) *ARF2* expression changes during pod development in wild diploid, wild tetraploid, and cultivated tetraploid lines. e) Allele frequency distribution of *ARF2‐A08* among 12 wild species and 58 cultivated lines that differ in pod size (small, median, and large).

Pod development directly affects peanut yield, and large pod is a major domestication trait. Cultivated peanuts have the pod size four to ten times higher than that of its tetraploid progenitor (**Figure** **4**a; and Figure S14, Supporting Information). To better understand the relationship between SV‐mediated expression changes and pod size domestication, we identified 18 putative seed development‐related genes, which are involved in cell elongation, cytokinin regulation, and cell division.^38^, ^39^, ^40^, ^41^, ^42^, ^43^, ^44^, ^45^, ^46^, ^47^, ^48^, ^49^, ^50^ These genes have SVs in upstream/exon regions (UERs) and most of them were expressed at higher levels in Hua8016 or Hua8017 than in *A. monticola* (Figure 4b; and Table S14, Supporting Information). AUXIN RESPONSE FACTORs (ARFs) play important roles in auxin‐mediated growth and development, including fruit and seed development.^45^, ^51^ *Arabidopsis MNT/ARF2*, an ortholog of peanut *ARF2*, is a negative regulator of seed size and weight by repressing cell division and organ growth.^45^ Peanut *ARF2* (*EVM0069298*) is located in chromosome A08 and has a 275 bp deletion and 7 bp insertion in the 12th exon in Hua8016 and Hua8017 (Figure 4c). While all 12 wild species with small pod size do not possess deletions in *ARF2*, the deletions are present in 25% of 58 cultivars surveyed with medium pod size (≤30 mm length) and 68% cultivars with large pod size (≥38 mm length) (Figure 4e; and Figure S15, Supporting Information). The SVs are found to be associated with different alternative splicing patterns of *ARF2* transcripts (Figure S16, Supporting Information), and expression level decrease in Hua8016 and Hua8017 at the development stages 1–3, relative to those in *A. monticola* (Figure 4d). It is likely that the low expression of *ARF2* due to the deletion/insertion may partially contribute to the increase in seed size in cultivated peanuts. Furthermore, some SV‐associated genes, such as *EVM0055972* and *EVM0047598*, encoding a vacuolar processing enzyme, coincide with the location of quantitative trait locus (QTLs) for pod weight and size in cultivated peanuts.^52^, ^53^ These data collectively suggest potential roles for SV‐associated genes in pod development and size selection.

### 2.6 Disease Resistance Genes in Wild Peanut

**Figure 5:**
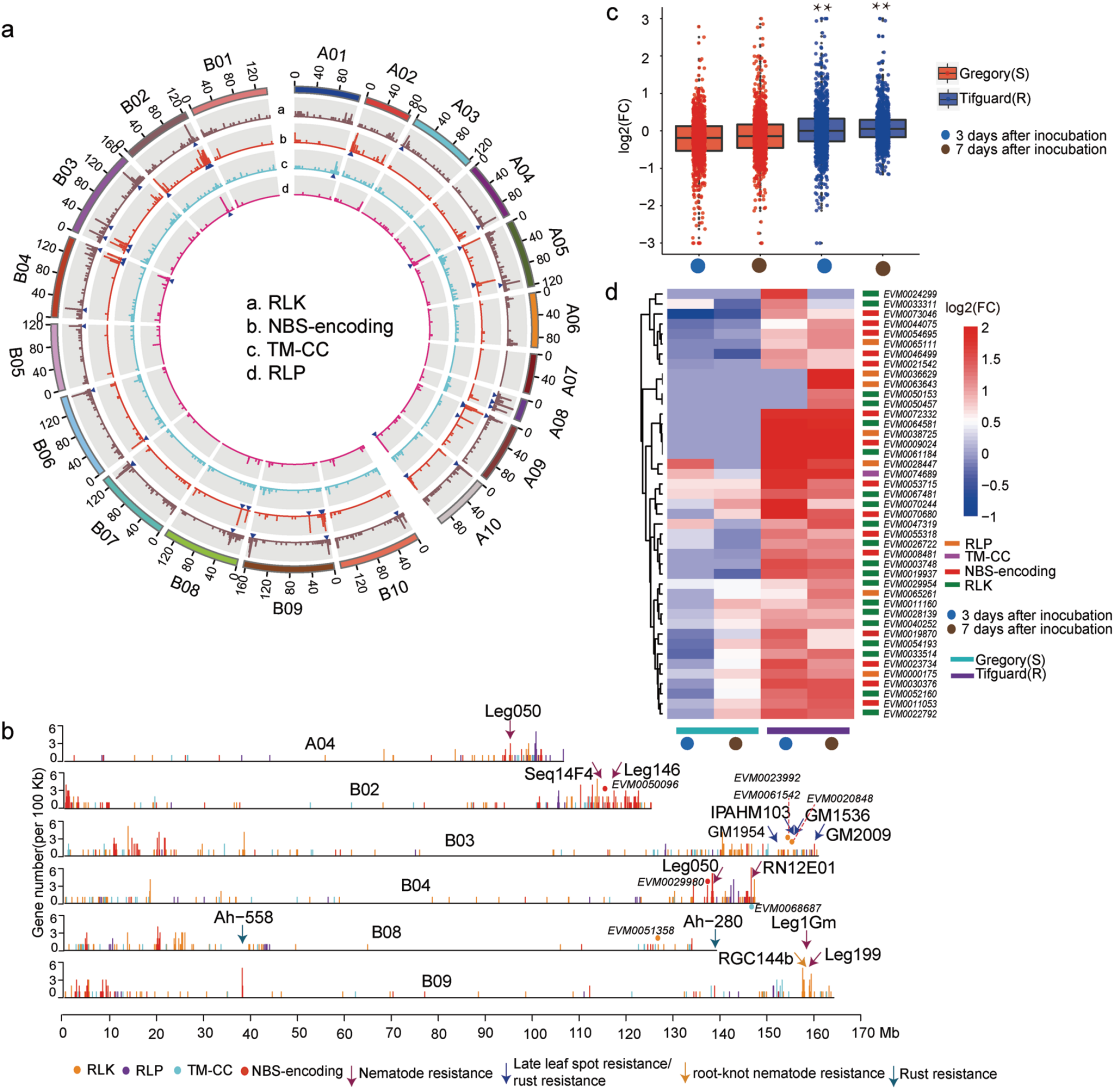
Characterization of resistance gene analogs (RGAs) in the wild peanut (*A. montocola*) genome. a) An overview of resistance genes in 500 kb nonoverlapping intervals. The histogram represents the number of genes. The blue arrows highlight the locations of RGA multi‐gene loci (more than five genes per 500 kb) on the pseudomolecules. b) Mapping of disease resistance QTLs to *A. monticola* genome. The placement of RGAs in each chromosome is displayed in 100 kb resolution (NBS‐encoding: red; TM‐CC: cyan; RLP: deeppink; and RLK: orange). Only QTLs adjacent to (one marker of QTL, <500 kb) or covered RGAs (two markers of QTL) are exhibited in figure. c) Expression pattern of R genes in root‐knot nematode infection roots. The fold change value is calculated by RPKM(infected)/RPKM(control). The blue and red panels represent root‐knot nematode susceptible and resistance groups, respectively. d) R genes in response to root‐knot nematode infection. R genes displaying equally expressed (fold change <= 2 or FDR >= 0.05) or scilent (RPKM = 0) in susceptible group but highly expressed (fold change >2 and FDR <0.05) in resistance group were considered. Labels of Gregory(S) and Tifguard(R) represent susceptible and resistance cultivated peanut lines, respectively. CN, CC–NBS; CNL, CC–NBS–LRR; NL, NBS–LRR; RLK, receptor‐like protein kinase; RLP, receptor‐like protein; TMCC, transmembrane coiled‐coil protein; TN, TIR–NBS; TX, TIR–unknown.

Disease is one of the most severe threats to peanut production.^54^ Using a disease resistance gene analog (RGA) prediction pipeline, we identified 2294 RGAs of nucleotide‐binding site (NBS), receptor‐like protein kinase (RLK), receptor‐like protein (RLP), and transmembrane coiled‐coil protein (TMCC) families in the *A. monticola* genome (Tables S15 and S16, Supporting Information). Notably, the NBS‐encoding genes, but not the remaining three classes of RGAs, are disproportionally more abundant in the distal regions of the chromosomes than in the proximal regions (**Figure** **5**a). RLPs are significantly enriched in A08 (Figure 5a), which may result from frequent rearrangement events between A08 and A07.^15^ QTLs are associated with resistance regions of nematode resistance,^55^, ^56^ root‐knot nematode (RKN) (Meloidogyne arenaria (Neal) Chitwood) resistance,^57^ rust resistance,^58^, ^59^ and late leaf spot resistance.^59^ Interestingly, we found 14 QTLs close to RGA‐enriched regions (Table S17, Supporting Information). For example, in chromosome A04, seven RGAs including two RLK genes and five NBS‐encoding genes are adjacent to the leg50 marker for nematode resistance (Figure 5b). Notably, two RLK gene copies of *EVM0023992* and *EVM0061542*, whose ortholog is leucine‐rich repeat transmembrane protein kinase (*AT4G20140*) in *A. thaliana*, located in IPAHM103‐GM1954 QTL region associated with late leaf spot resistance and rust resistance (Figure 5b). In Hua8106 and Hua8107 accessions, *EVM0023992* and *EVM0061542* have a deletion variation in their upstream and exon regions, respectively. *EVM0068687*, encoding a transmembrane coiled‐coil protein, is associated with RN12E01 marker of nematode resistance QTL in 146.26 Mb of chromosome B04. We found an insertion variation in exon region of *EVM0068687* in both Hua8106 and Hua8107 (Figure 5b).

*Meloidogyne arenari*a is the most prominent pathogen to the cultivated peanut industry as its wide distribution in the peanut production regions.^60^ Resistance alleles to RKN in chromosome A09 of a wild peanut relative (*A. cardenasii*) were introgressed into cultivated peanut.^61^ Scanning the transcriptome data of *M. arenaria* resistance experiments with root‐knot nematode infected plants at 0, 3, and 7 d after inoculation for susceptible and resistance groups showed that fold change (FC) values of RGAs are significantly higher in resistance group (Wilcoxon signed‐rank test, *P* <= 0.01) (Figure 5c).^62^ We found that 43 RGAs showed induction after inoculation in high resistance group, while displayed no expression changes or repression in susceptible group (Figure 5d). These candidate *M. arenaria* resistance genes occupied 1.87% of RAGs, which is nearly five times higher than the ratio of 276 candidates (0.37%) in genome‐wide scale. Among them, NBS and RLK family members are primarily disease‐resistance genes being positive response to root‐knot nematode infection.^63^, ^64^ Further, we identified 190 RGAs with SVs (SV‐RGAs) in *A. hypogaea* (Table S18, Supporting Information). SV‐RGAs had higher expression change in high resistance group, especially in 7 d after inoculation (Wilcoxon signed‐rank test, *P* <= 0.01) (Figure S17, Supporting Information). Among these SV‐RGAs, 32 genes showed significant expression changes in susceptible or resistance group after inoculation (Figure S18, Supporting Information). The proportion of differentially expressed SV‐RGAs (16.8%) is higher than the differentially expressed RGAs (11.2%). These results indicate the potential role of SVs in RKN resistance. As we know, some alleles associated with high resistance traits had been lost in modern peanuts. The alleles present in the wild *A. monticola* provide a genetic resource for introducing pest and disease resistance into peanut breeding programs.

## 3 Discussion

Cultivated peanuts were domesticated from the wild tetraploid *A. monticola*, which was formed between two diploid species *A. duranensis* and *A. ipaensis*.^3^, ^4^, ^5^, ^6^, ^7^, ^8^ While genome sequences of wild diploids, wild tetraploid, and cultivated tetraploid species of peanuts are available, little is known about subgenome evolution and trait domestication in tetraploid peanuts.^2^, ^15^, ^16^ Sequencing and comparative analyses of the wild tetraploid species have filled the genomic and evolutionary gap between the wild diploids and cultivated tetraploids. Using the *A. monticola* sequences, we reconstructed the allotetraploid evolution model and found the monophyletic origin of A and B subgenomes of *A. hypogaea*. After polyploid formation, A and B subgenomes are subjected to asymmetric homoeologous sequence exchanges, gene family expansion and contraction, homoeolog expression divergence, and selection. Notably, natural selection biases toward SV genes of B subgenome in wild *A. monticola*, but domestication has larger effects on SV genes of A subgenome in cultivated *A. hypogaea*. This difference does not seem to correlate with two diploid ancestral genomes; the B‐ancestral genome is larger and underwent more local changes than the A‐ancestral genome in diploids.^15^ Asymmetrical evolution in cotton is likely associated with the size difference between two subgenomes, in which A‐ancestral genome is twice the size of the D‐ancestral genome.^12^ In alloploids like *B. napus* 13 or *B. juncea* 30 with similar ancestral genomes, HSEs and expression divergence dominate genetic and genomic diversity after polyploid formation. We found that the A‐subgenome orthologous proteins of *A. monticola* were distinctly less similar to *A. duranensis* than the B‐subgenome, suggesting higher genomic diversity in A‐genome.^15^ In peanuts, although the cause of natural selection on the wild tetraploid and domestication on cultivated tetraploids is unknown, our data provide the evidence for SVs contributing to asymmetrical subgenome evolution and homoeolog expression divergence in wild and cultivated tetraploid peanuts. SV‐associated genes are subject to natural selection and human domestication, which may affect agronomic traits such as pod size and development. In cotton, asymmetric subgenome evolution are found to be related to fiber traits.^65^ Low levels of resistance to pests and disease in cultivated species significantly reduce peanut grain quality and yield worldwide.^64^ Wild relatives remained rich genetic diversity and high levels of resistance to many pathogens. The accurate identification and characterization of NBS‐leucine rich repeats (LRRs) and other complex resistance gene families and QTLs in wild *A. monticola* should substantially contribute to the repertoire of resistances and improve peanut production. Sequence and comparative genomic resources of wild and cultivated tetraploid peanuts have provided new information for illuminating our understanding of evolution and domestication of polyploid crops, as well as genomic tools for improving agronomic traits of this polyploid crop with global importance on economy and food security.

## 4 Conclusions

This high‐quality sequence of wild tetraploid peanut has filled a genomic and evolutionary gap between diploid and cultivated tetraploid species and provided evidence for a monophyletic origin of A and B subgenomes. Comparative analyses of diploid ancestral species, as well as wild and cultivated tetraploid species, have revealed a role for asymmetrical evolution of A and B subgenomes, especially SV‐associated genes in pod size domestication. These genomic resources are uniquely valuable for studying polyploid genome evolution, crop domestication, and genome‐assisted improvement of peanut production.

## 5 Experimental Section

*Plant Materials and Genome Assembly*: Peanut plants were grown in a growth chamber at 25 °C. The genomic DNA of *A. monticola* was extracted from fresh leaves of 30 d old wild peanut seedlings (Line PI 263393) using DNeasy Plant Mini Kit (Qiagen, Beijing, China). The genome was assembled using integrated strategies including paired‐end and mate‐paired libraries reads range from 100 bp to 17 kb fragments, single molecule real time (SMRT) sequencing, BioNano optics, and Hi‐C. RNA‐seq data from pooled tissues of leaves, stems, roots, and pods of *A. monticola* were generated for assistance in annotating gene models.

Seed samples in different developmental stages (Stage 1: ≈15 days after flower (DAF); Stage 2: ≈30 DAF; Stage 3: ≈50 DAF; and Stage 4: ≈70 DAF) were collected for RNAs extraction. RNA‐seq and full‐length isoform sequencing was made from two diploid ancestors including *A. duranensis* and *A. ipaensis*, wild tetraploid *A. monticola* (small pod), and cultivated peanut *A. hypogaea* (median pod of Hua8106 and large pod of Hua8107 from two sister lines, respectively). Meanwhile Hua8106 and Hua8107 were preformed whole genome resequencing with high depth.

Fresh leaves of 17 wild diploid accessions within AA, CC, EE, AmAm, ExEx, E3E3 peanut sections, three wild allotetraploid accessions of *A. monticola*, and seven cultivated accessions of *A. hypogaea* within AABB sections were collected for DNA preparation in whole genome resequencing. Other 20 cultivated accessions were downloaded from NCBI in BioProject accessions PRJNA340877.

*Repetitive elements (TE) Annotation*: Homolog and de novo strategies were both applied to identify repetitive sequences in the wild peanut genome. Software, including RepeatScout v1.0.5,^66^ LTR‐FINDER v1.05,^67^ MITE‐hunter‐20100819,^68^ and PILER‐DF v1.0,^69^ was used for ab initio prediction. The results obtained from software were combined to form a new repetitive sequence database. This database was then merged with Repbase v19.06^70^ and classified into different categories by the PASTEClassifier.py^71^ script included in REPET v2.5.^72^ Repetitive sequences in the wild peanut genome were identified by homolog searching with the final merged database through RepeatMasker v4.0.5.^73^

*Gene Prediction and Annotation*: De novo, homology based, and transcriptome‐based strategies were applied to predict protein‐coding genes in the wild peanut genome. Three pieces of software, including Genscan (http://genes.mit.edu/GENSCAN.html), Augustus v2.4,^74^ and GlimmerHMM v3.0.4,^75^ were used for de novo prediction. The homolog‐based prediction was refined by GeneWise v2.4.1 and GeMoMa v1.3.1.^76^, ^77^ Transcriptome data that were generated from pooled tissues of leaf, stem, and root of wild peanut were mapped and assembled using Hisat v2.0.4^78^ and Stringtie v1.2.3,^79^ respectively. Unigenes were aligned to the genome assembly using BLAT^80^ and then filtered using PASA2.0.4.^81^ Pooled transcriptome data were also mapped to the reference genome using TopHat^82^ and transcripts assembled with Cufflinks.^83^ Transdecoder v2.0^84^ was then applied to identify the gene structure of new gene models and transcripts derived from Cufflinks. Predicted gene structures were integrated into consensus gene structures using EVidenceModeler v1.1.1.^85^ The completeness of predicted genes was assessed using BUSCO analyses with eudicotyledons database (https://busco.ezlab.org/).

*Pseudogenes Annotation*: Proteins of *A. monticola* were aligned to its genome with masking predicted functional genes using GenBlastA v1.0.4.^86^ Pseudogenes were then identified via GeneWise V2.4.1^76^ from these candidate homolog regions, which had a frame shift and/or premature stop code occurrence in the coding region.

*Full‐Length Isoform Sequencing and Analysis*: Full‐length isoform sequencing was conducted in two diploid ancestors (small pod of A.du and A.ip) including *A. duranensis* and *A. ipaensis*, wild tetraploid *A. monticola* (small pod of A.mon) and cultivated peanut *A. hypogaea* (large pod of Hua8107), respectively. Peanut plants were grown in a growth chamber at 25 °C. The pod samples were collected from the third stage of pod development (Stage 3: ≈50 DAF), which exhibited the most apparent pod size difference during pod development. RNAs were extracted from pod tissues using the RNeasy Plus Mini kit DP441 (Qiagen, Beijing, China). The size fractions of cDNA (1–6 kb) after five cycles of polymerase chain reaction (PCR) were collected from a 0.8% agarose gel. After size selection, these cDNA fractions were treated with DNA damage repair mix, followed by end repair and ligation of SMRT adapters using the PacBio SMRTbell Template Prep Kit to create PacBio libraries. These four size‐fractional libraries were sequenced on the Sequel platform. Raw reads were processed into error‐corrected reads of insert (ROIs) using Iso‐seq pipeline. Then, the ROIs were classified into circular consensus sequences (CCS) and non‐CCS subreads by ToFu v2.3.0 based on presence or absence of sequencing adapters.^87^ Full‐length and nonchimeric transcripts were determined by each having both the primer sequences and the polyA tail signal in ROIs. Then, a clustering algorithm, Iterative Clustering for Error Correction, was used to get consensus transcripts for all full length (FL) transcripts. Quiver (PacBio) was used to polish the consensus transcripts to give rise to the high‐quality FL transcripts with more than 99% post‐correction accuracy.

*lncRNA Identification from Isoform Sequencing*: Four computational approaches that include CPC/CNCI/CPAT/Pfam were combined to sort nonprotein coding RNA candidates from putative protein‐coding RNAs in the transcripts. lncRNA candidates were defined as those with transcript length more than 200 nt and more than two exons. These candidates were further distinguished using CPC/CNCI/CPAT/Pfam for potential protein coding possibility assessment.

*Noncoding RNA Prediction*: tRNAscan‐SE v2.0 was applied to tRNA detection and functional prediction.^88^ miRNAs were identified by homolog searching with one mismatch allowed using miRBase (Release 22) as a reference.^89^ The second structures of putative sequences were predicted by miRDeep2.^90^ Other ncRNAs were predicted by software Infernal v1.1.2 using default parameters.^91^ The family of ncRNA was identified based on database Rfam v12.1.^92^

*DNA Preparation and Sequencing*: Genomic DNA of diploid and tetraploid peanut accessions was extracted from fresh leaves using DNeasy Plant Mini Kit (Qiagen, Beijing, China). After quality control by Agilent 2100 Bioanalyzer and real‐time quantitative PCR detecting (qPCR) system, all the PCR‐free libraries were sequenced on an Illumina X‐TEN platform with 150 bp paired‐end sequencing strategy.

*Population SNPs Detection*: Short read sequencing data of 20 cultivated accessions were downloaded from NCBI in BioProject accessions PRJNA340877. Seventeen wild diploid accessions within AA, CC, EE, AmAm, ExEx, and E3E3 peanut sections, three wild allotetraploid accessions of *A. monticola*, and seven cultivated accessions of *A. hypogaea* were also collected and sequenced. All clean reads of tetraploid accessions were mapped to the combined reference genome of diploid ancestors as *A. duranensis* (A subgenome) and *A. ipaensis* (B subgenome) using BWA v0.7.17 with default parameters.^93^ Clean reads of each tetraploid accession were separated into two putative A and B diploid groups according to their alignment results. All diploid accessions including putative accessions driving from tetraploid accessions were mapped to the reference genome of diploid ancestors as *A. duranensis* (A subgenome) and *A. ipaensis* (B subgenome), respectively, using BWA v0.7.17 with default parameters.^93^ The Picard tools v1.9.4 (https://broadinstitute.github.io/picard/) were used to sort the alignment result sequence alignment/map (SAM) files. SNPs and indels were called using Genome Analysis Toolkit.^94^ Only SNPs with the minors allele frequency >0.05 and minimum integrity >0.5 were retained for further analyses.

*Population Phylogenetic Relationship Construction*: SNPs with full integrity and located at protein coding region were selected for population phylogenetic relationship construction. HKY85 model in PhyML‐20151210 was applied to construct the maximum likelihood (ML) tree with 1000 bootstrap replicates.^95^

*Principal Component Analysis*: The PCA was proceeded using SMARTPCA within the EIGENSOFT v6.0 packages with default parameters.^96^

*Identification of Syntenic Orthologs and Homoeologs*: The longest transcript was selected to represent the corresponding protein coding gene. The syntenic gene pairs between genomes of two species were assessed by aligning the proteins to each other using blastp with E‐value <1e‐5.^97^ Synteny blocks between each other were called using McScanX with default parameter.^98^ Only synteny blocks having more than five gene pairs were considered. All gene pairs within a syntenic block were considered as syntenic orthologs between genomes of two species. For identification of homoeologous gene pairs between subgenome of *A. monticola*, similar strategy was applied for syntenic block inspection between two subgenomes.

*Whole Genome Duplication Events*: Self‐alignment of protein sequences using blastp with E‐value <1 × 10^–5^ was carried out.^97^ Internal syntenic blocks (regions with at least five collinear genes) were identified using MCScanX with default parameter.^98^ *Ks* values of paralogous gene pairs were calculated using the yn00 program from the PAML package.^99^ The peak of *Ks* distribution derived from internal paralogous gene pairs was considered as whole genome duplication events.

*Time Estimation for Allotetraploid Formation*: Synonymous substitution rates (*Ks*) of syntenic orthologs between A subgenome of *A. monticola* and its ancestor *A. duranensis* as well as B subgenome and its ancestor *A. ipaensis* were calculated. The divergence time between subgenome and its ancestor was estimated as formula: *T* = peak *Ks* / 2*m, in which *T* means divergence time, peak *Ks* represents the *Ks* value of gene pairs with highest frequency, and *m* means molecular clock. The average rates of change for *Arachis* as 8.12 × 10^−9^ mutations per base per year was considered, which is supported by the previous work.^15^ Notably, the *Arachis* lineages have been accumulating silent changes relatively quickly (≈1.4 times faster) since the divergence of the Dalbergioid clade.^15^ Thus, the age of early whole genome duplication was directly referred from previous study.^100^, ^101^

*Phylogenetic Tree Construction and Diverge Time Estimation*: A total of 151 single‐copy orthologs were obtained through gene family cluster using OrthoMCL v2.0.9.^102^ The protein sequences of single‐copy orthologs were aligned by MUSCLE v3.8.31 and then concatenated into a super‐gene sequence.^103^ Then the phylogenetic tree was constructed using the ML algorithm with the JTT amino acid substitution model implemented in phyML‐20151210 software. The divergence time was estimated using the MCMCtree program in PAML v4.7b (Phylogenetic Analysis of ML) package.^99^ Five calibration points (root: 93–106 MYA, *Vigna angularis* vs *Proteus vulgaris* (*P. vulgaris*): 9.10–10.40 MYA, *Trifolium subterraneum* vs Medicago *truncatula*: 24.70–29 MYA, Glycine max (*G. max*) vs *P. vulgaris*: 23.82–24 MYA and *A. duranensis* vs *A. ipaensis*: 2.15–2.17) were applied to constrain the divergence time of the nodes.

*Gene Family Evolution*: The OrthoMCL^102^ methodology was used to cluster gene families and then the CAFE v2.2^104^ package was applied to identify the expanded and contracted gene family (*P* < 0.01).

*HSEs between Two Subgenomes*: The HSEs were identified according to previous method successfully applied in *B. napus* genome project.^13^ Briefly, segmental HSEs were revealed based on sequence read coverage from allotetraploid wild and cultivated peanuts. The average depth was calculated based on 10 kb nonoverlapping window. The windows whose read coverage was between 1.5 times and 4 times greater than the whole genome average depth were identified as regions of the parental genomes that displayed double coverage. The double coverage windows were considered as candidate duplications and regions with low or no coverage were regarded as deletions. The distance at most five adjacent windows with depth greater than the threshold was linked together. Only regions spanning more than eight windows (80 kb) were retained as candidate HSEs.

*Ka/Ks Calculation*: To assess the selection bias for subgenomes, dominant genes, and SV genes, average nonsynonymous/synonymous substitution (*Ka*/*Ks*) value (ω = *Ka*/*Ks*) was estimated using the YN00 program of PAML v4.2b package with default parameters.^99^ The natural selection pressure of wild tetraploid *A. monticola* was estimated by *Ka*/*Ks* between *A. monticola* and its ancestors. The human selection pressure of cultivated tetraploid *A. hypogaea* was estimated by *Ka*/*Ks* between *A. hypogaea* and *A. monticola*.

*Identification of SVs*: Resequencing with high coverage (>40x) was made from two representative cultivated lineages of *Hua8106* with median pod and *Hua8107* with large pod, and two ancestors with small pod. Clean reads were mapped onto the reference genome of *A. monticola* using BWA v0.7.17 software with default parameters.^93^ For sequencing data of two diploid ancestors, these were mapped to corresponding A and B subgenomes, respectively. BreakDancerMax‐0.0.1r61 was used for genome‐wide detection of structural variants (inversion, deletion, insertion, intra‐chromosome translocation, and inter‐chromosome translocation) from next‐generation paired‐end sequencing reads with default parameters.^105^ To control the false SVs, the confident SVs were further filtered through the split‐alignment reads across the breakpoint of SV. Clipped alignment reads (denotes as “S” in CIGMA column of SAM file) were extracted from bam file around the 300 bp distance of SV breakpoint position. Split read with more than 10 bp soft‐clipped sequences was considered as confident split read, which aligned across breakpoint. If breakpoint of SV supported by more than five confident soft‐clipped reads, the SV was considered as confident one. Deletion and insertion structure variations less than 5 bp or more than 10 kb would be discarded. For each pair of homoeologs between A and B subgenomes (see details in the *Identification of Syntenic Orthologs and Homoeologs* section), one gene was defined with SVs in its upstream or gene body regions as SV‐gene and the other gene without SVs in its upstream or gene body regions as non‐SV gene. Both of genes in a homoeologous pair without SVs in their upstream or gene body were defined as unaffected genes.

*Tandem insertion identification*. The tandem insertion was defined according to the origin of clipped sequence (unmapped) of soft‐clipped reads across breakpoint of insertion as example of 5′ soft‐clipped read (86S64M) and 3′ soft‐clipped read (86M64S) (Figure S11a, Supporting Information). Clipped subsequence (defined as flag) of soft‐clipped reads was remapped onto the reference genome using bowtie2 alignment software with end‐to‐end parameter. If the matched locus of flag located around the 10 kb distance of breakpoint, the insertion locus was defined as tandem insertion event (Figure S11a, Supporting Information).

*Inspection of insertion origin*. Two types of soft‐clipped read (example of “86S64M” and “86M64S”) across the breakpoint of insertion would be produced in reads mapping (Figure S11b, Supporting Information). The origin of insertion locus was inspected according matched locus of paired clipped‐sequences (flag sequences). Paired flags of paired soft‐clipped‐reads were extracted and remapped onto the reference genome using bowtie2 alignment software with end‐to‐end parameter. If paired flags both matched in same chromosome (similar to paired end alignment) and the distance was less than 10 kb, the corresponding matched locus was considered as origin locus of this insertion event and defined as intra‐chromosome insertion event. If paired flags both matched in different chromosome (similar to paired end alignment) and the distance between two flags was less than 10 kb, the corresponding matched locus was considered as origin locus of this insertion event and defined as inter‐chromosome insertion event (Figure S11b, Supporting Information).

*mRNA Preparation and Sequencing for Pod Development*: RNA‐seq was made from two diploid ancestors (small pod) including *A. duranensis* and *A. ipaensis*, wild tetraploid *A. monticola* (small pod) and cultivated peanut *A. hypogaea* (median pod of Hua8106 and large pod of Hua8107), respectively. Peanut plants were grown in a growth chamber at 25 °C. Developmental stages of peanut pods during initiation and maturation were determined according to previous report.^106^ Pod collection was performed for four pod development stages (Stage 1: ≈15 DAF; Stage 2: ≈30 DAF; Stage 3: ≈50 DAF; Stage 4: ≈70 DAF), which exhibited apparent pod size difference. Three biological replicates were designed for RNA‐seq experiments. RNAs were extracted from pod tissues using the RNeasy Plus Mini kit DP441 (Qiagen, Beijing, China), and then sequenced on the Illumina HiSeq sequencing platform.

*Transcriptome Analysis*: Clean reads were mapped to wild peanut reference genome by TopHat v2.0.13.^107^ Gene expression levels were calculated by Cufflinks v2.2.1 using default parameters.^83^ The gene expression levels were normalized by reads per million per kilo bases (RPKM). A gene with an expression value greater than 0.1 RPKM was considered as expressed.^108^ Differentially expressed genes (DEGs) were identified by DEseq2 package and a *t*‐test with Benjamini–Hochberg (BH) multiple hypothesis testing correction.^109^, ^110^ Cutoff values for DEGs were FC larger than 2 or smaller than 0.5, and false discovery rate (FDR) < 0.05.

*Identification of Dominantly, Under‐Dominantly, and Equally Expressed Genes*: Homoeolog expression dominance analysis was performed within homoeologous gene pairs between A and B subgenomes for wild and cultivated tetraploid peanuts, respectively. To identify the significance of a gene expression bias between homoeologous gene pairs, a *t*‐test with BH multiple testing correction was used.^110^ For each homoeologous gene pair, if the fold change of expression levels was >2 and FDR < 0.01 in at least one stage of pod development and expression pattern was consistent in other stages, this gene pair was defined as homoeologous expression dominant. Between two copies of a homoeologous gene pair, the copy that was expressed at higher levels than the other copy was defined as dominant, and the other copy as under‐dominant. The two copies that showed no expression difference were defined as equally expressed or neutral.

*PCR Validation of Deletion in ARF2‐A08 Locus*: Seventy peanut accessions with diverse pod phenotypes including 18 diploid ancestors and tetraploid of small pod (*A. duranensis*, *A. duranensis*, and *A. monticola*) 24 cultivated accessions of median pod, and 28 cultivated accessions of large pod for *ARF2‐A08* deletion genotype validation were collected. Total DNA was extracted from young leaves (*A. duranensis*, *A. duranensis*, *A. monticola*, *Hua8106*, and *Hua8107*) using DNeasy Plant Mini Kit (Qiagen, Beijing, China) and diluted to ≈100 ng µL^−1^. Primers (F: ACGGAGGTACGGTTCAGAGA; R: CGAGCATCATGTCACCCTCA) for ARF2‐A08 were designed based on the 12th exon including 275 bp deletion. The 25 µL PCR reactions contained final concentrations of 0.05 U µL^−1^ PrimeSTAR HS DNA Polymerase, 5 µL 5 × PrimeSTAR Buffer (Mg2+ Plus), 2 µL 1 × dNTP Mixture (2.5 × 10^−3^ m each), 1 µL 100 × 10^−6^ m each Primer, 5 ng µL^−1^ DNA template. Reactions were cycled on a thermal cycler (94 °C 3 min, 30 cycles of [94 °C 40 s, 52 °C 40 s, 72 °C 30 s], 72 °C 10 min) and the amplification products were separated using agarose gel electrophoresis and examined on a SYSTEM GelDoc XR+ (Bio‐Rad, California, USA).

*Analysis of RGAs*: RGAs of two ancestor (*A. duranensis* and *A. ipaensis*), wild peanut (*A. monticola*) were predicted using RGAugury pipeline,^42^ which integrated domain prediction software Hmmer3,^111^ Pfam,^112^ COILS (with a minor modification),^113^ and interproscan.^114^ The genomic location cluster of resistance genes was defined by more than five genes in 500 kb nonoverlapping window for NBS‐coding, RLK, TM‐CC, and RLP class, respectively. A total of 28 transcriptome accessions of *M. arenaria* infected experiments from root‐knot nematode infected plants at 0, 3, and 7 d after inoculation were obtained from previous publication.^62^ The gene expression level (RPKM) and different expression genes were analyzed as described in the *Transcriptome Analysis* section. The expression difference of RGAs between infected and control groups of high resistance Tifguard(R) and susceptible Gregory(S) peanuts was shown in heatmap with log2 (fold change) transformation.

## Conflict of Interest

The authors declare no conflict of interest.

## Supporting information

Supporting Information

Supporting Information
