# Supplementary material for: Comparison of Arachis monticola with Diploid and Cultivated Tetraploid Genomes Reveals Asymmetric Subgenome Evolution and Improvement of Peanut
Source: Adv Sci (Weinh). 2019 Nov 28;7(4):1901672. doi: 10.1002/advs.201901672 (PMC7029647; doi:10.1002/advs.201901672)
Supplement: Supplementary file 1 — Supporting Information [file ADVS-7-1901672-s001.pdf]

Copyright WILEY-VCH Verlag GmbH & Co. KGaA, 69469 Weinheim, Germany, 2019.

ADVANCED SCIENCE
Open Access

Supporting Information

for *Adv. Sci.,* DOI: 10.1002/advs.201901672

Comparison of *Arachis monticola* with Diploid and Cultivated Tetraploid Genomes Reveals Asymmetric Subgenome Evolution and Improvement of Peanut

Dongmei Yin, Changmian Ji, Qingxin Song, Wanke Zhang, Xingguo Zhang, Kunkun Zhao, Charles Y. Chen, Chuantang Wang, Guohao He, Zhe Liang, Xingli Ma, Zhongfeng Li, Yueyi Tang, Yuejun Wang, Ke Li, Longlong Ning, Hui Zhang, Kai Zhao, Xuming Li, Haiyan Yu, Yan Lei, Mingcheng Wang, Liming Ma, Hongkun Zheng, Yijing Zhang, Jinsong Zhang,* Wei Hu,* and Z. Jeffrey Chen**

Copyright WILEY-VCH Verlag GmbH & Co. KGaA, 69469 Weinheim, Germany, 2019.

Supporting Information

Comparison of *Arachis monticola* with diploid and cultivated tetraploid genomes reveals asymmetric subgenome evolution and improvement of peanut

Dongmei Yin, Changmian Ji, Qingxin Song, Wanke Zhang, Xingguo Zhan, Kunkun Zhao, Charles Y. Chen , Chuantang Wang, Guohao He , Zhe Liang, Xingli Ma, Zhongfeng Li, Yueyi Tang, Yuejun Wang, Ke Li, Longlong Ning, Hui Zhang, Kai Zhao, Xuming Li, Haiyan Yu, Yan Lei, Mingcheng Wang, Liming Ma, Hongkun Zheng, Yijing Zhang, Jinsong Zhang*, Wei Hu* and Z. Jeffrey Chen**

Supplementary figures

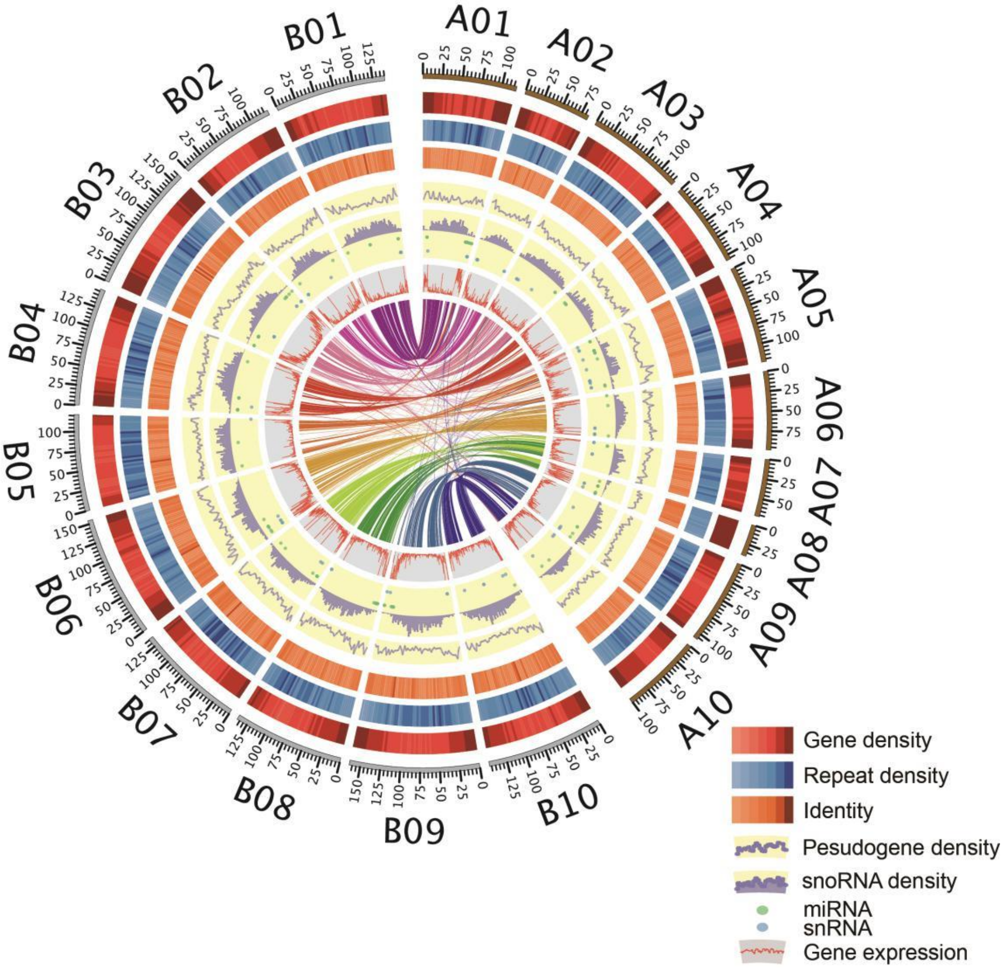

Supplementary Fig. 1 Genome-wide view and synteny between A and B subgenomes of *A. monticola*. Tracks indicate from outside to inside: gene density, repeat sequences, sequence identity estimated from protein sequences of syntenic ortholog pairs between tetraploid and their ancestors, pesudogenes, small nucleolar RNAs, microRNAs and small nuclear RNAs, genome-wide gene expression, and synteny relationship between A and B subgenomes. The non-overlapping sliding window is 3 Mb. The *A. monticola* genome has ~73.2% of transposable elements (TEs) including

the most abundant LTR/Gypsy (45.4%) and PLE|LARD (19.9%) elements (Table S1). The genome has 11,569 pseudogenes with frame shift and/or premature stop codon (Table S2). We also identified 15,431 non-coding RNAs (ncRNAs), occupying 1.91 Mb of *A. monticola* genome (Table S3). The genomic landscape of TEs and pseudogenes is similar in the two subgenomes, forming dense accumulation in pericentromeric regions. Genome-wide pooled transcriptome data showed high active transcription near chromosome ends, consistent with the gene density distribution.

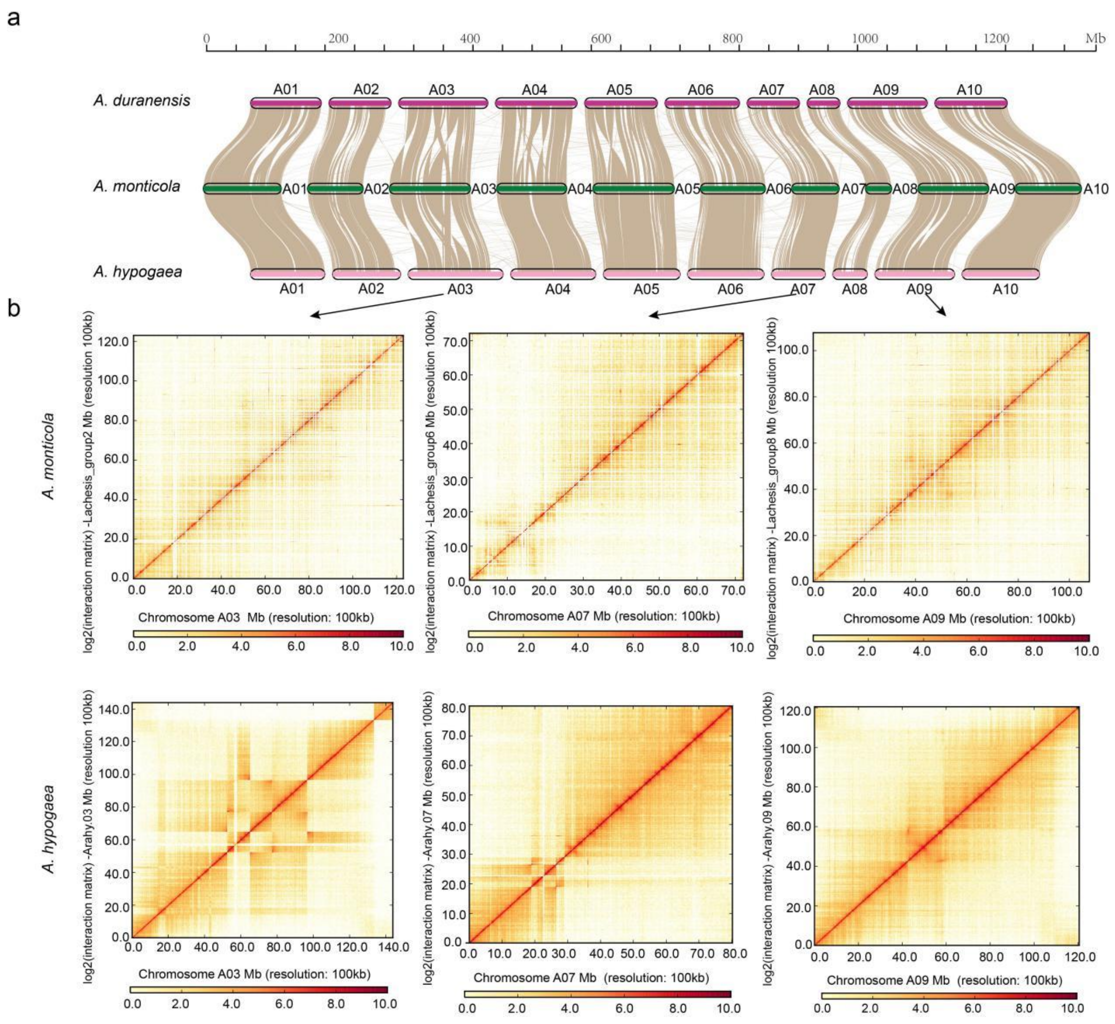

Supplementary Fig. 2 Genome comparison analysis within three A subgenomes. (**a**) Direct comparative genome analysis among three A genomes of diploid ancestor, wild tetraploid and cultivated tetraploid. (**b**) Top panels represent mapping Hi-C data of A. monticola against itself genome sequences (A.mon) and bottom panels show a chromatin interaction matrix of A. monticola against the A. hypogaea genome (cv. Tifrunner). The Hi-C heatmaps are showed at 100 kb resolution.

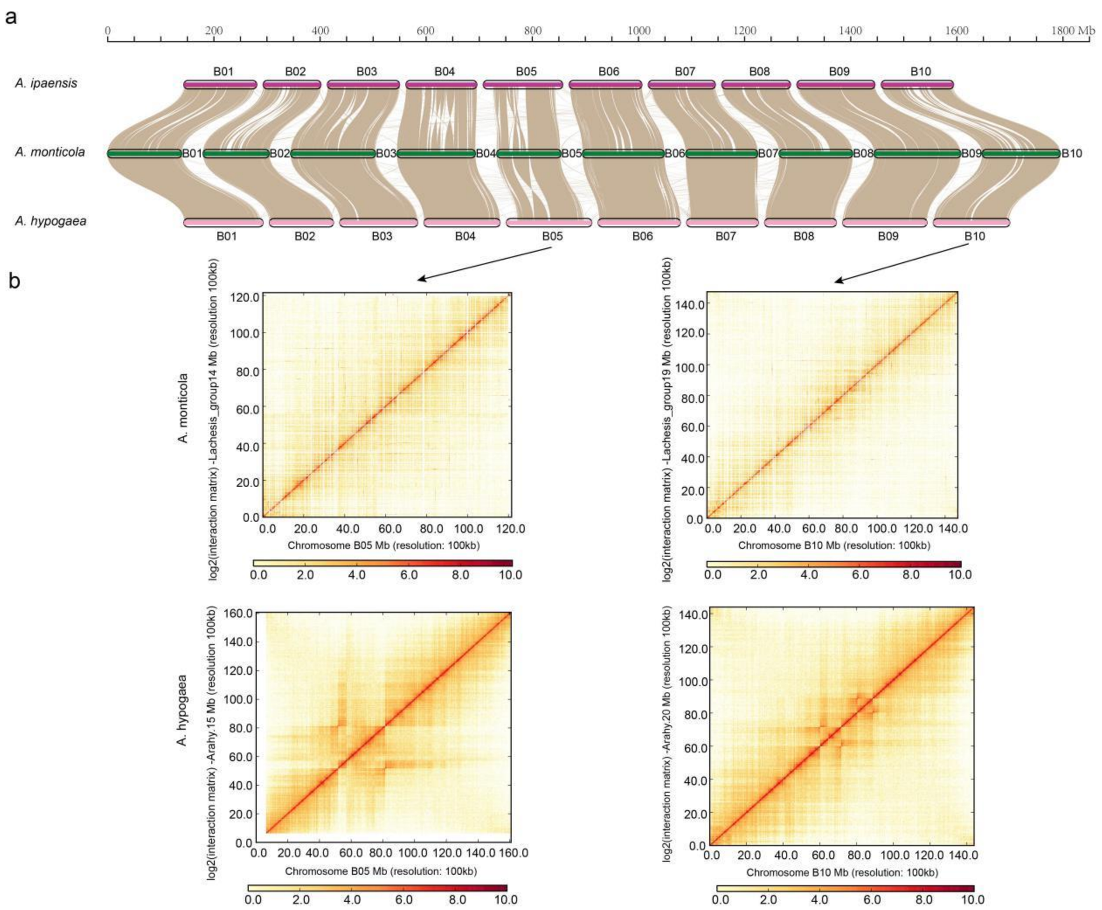

Supplementary Fig. 3 Genome comparison analysis within three B subgenomes. (**a**) Direct comparative genome analysis among three B genomes of diploid ancestor, wild tetraploid and cultivated tetraploid. (**b**) Top panels represent mapping Hi-C data of A. monticola against itself genome sequences (A.mon) and bottom panels show a chromatin interaction matrix of A. monticola against A. hypogaea genome (cv. Tifrunner). The Hi-C heatmaps are showed at 100 kb resolution.

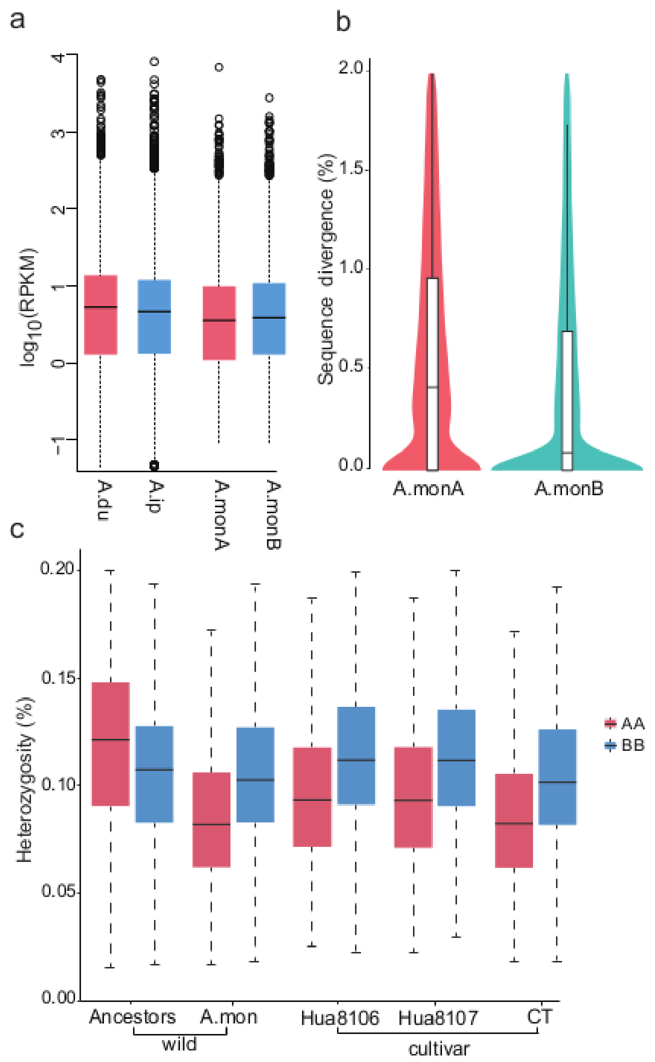

Supplementary Fig. 4 Whole genome comparison between tetraploid and its ancestors. (**a**) Genome-wide distribution of expression in *A. monA*, *A. monB* and their ancestors. Only syntenic gene pairs were considered. Expression level was calculated using pooled transcriptome data of root, stem, leaf, and flower. Pooled transcriptome data of progenitors were downloaded from NCBI (*A. duranensis*: SRS672917 and *A. ipaensis*: SRS672918). (**b**) Sequence divergence between *A. monA* and *A. duranensis*, and *A. monB* and *A. ipaensis* (Wilcoxon rank-sum test, $P < 0.01$). (**c**) Heterozygosity

distribution of different peanut lines. The heterozygosity rate was estimated using 100-kb non-overlapping window.

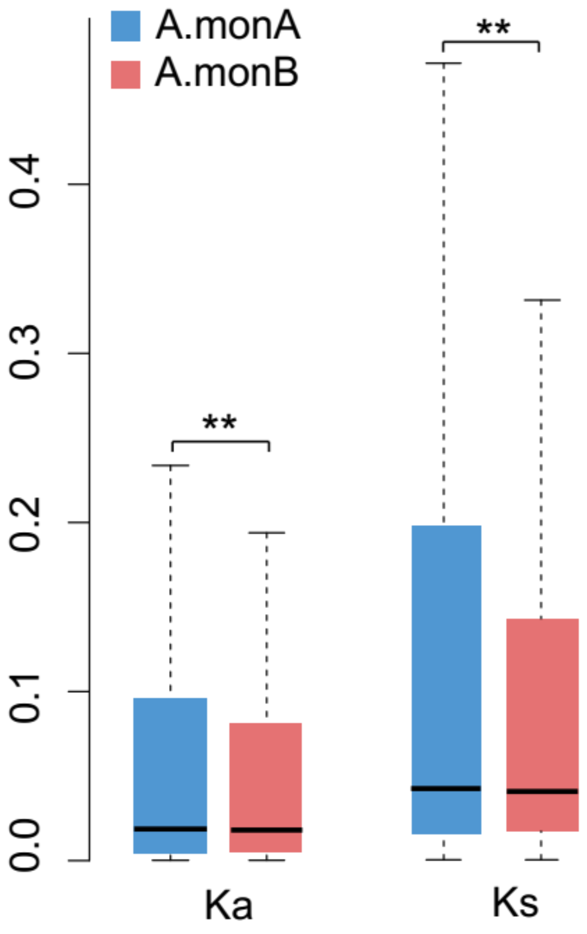

Supplementary Fig. 5 Ka and Ks values distribution between *A. momA* and *A. monB*. The values were calculated between syntenic orthologs of subgenomes and their ancestors. *A. monA* accumulated more mutations than *A. monB* (Wilcoxon rank-sum test, ***P* < 0.01).

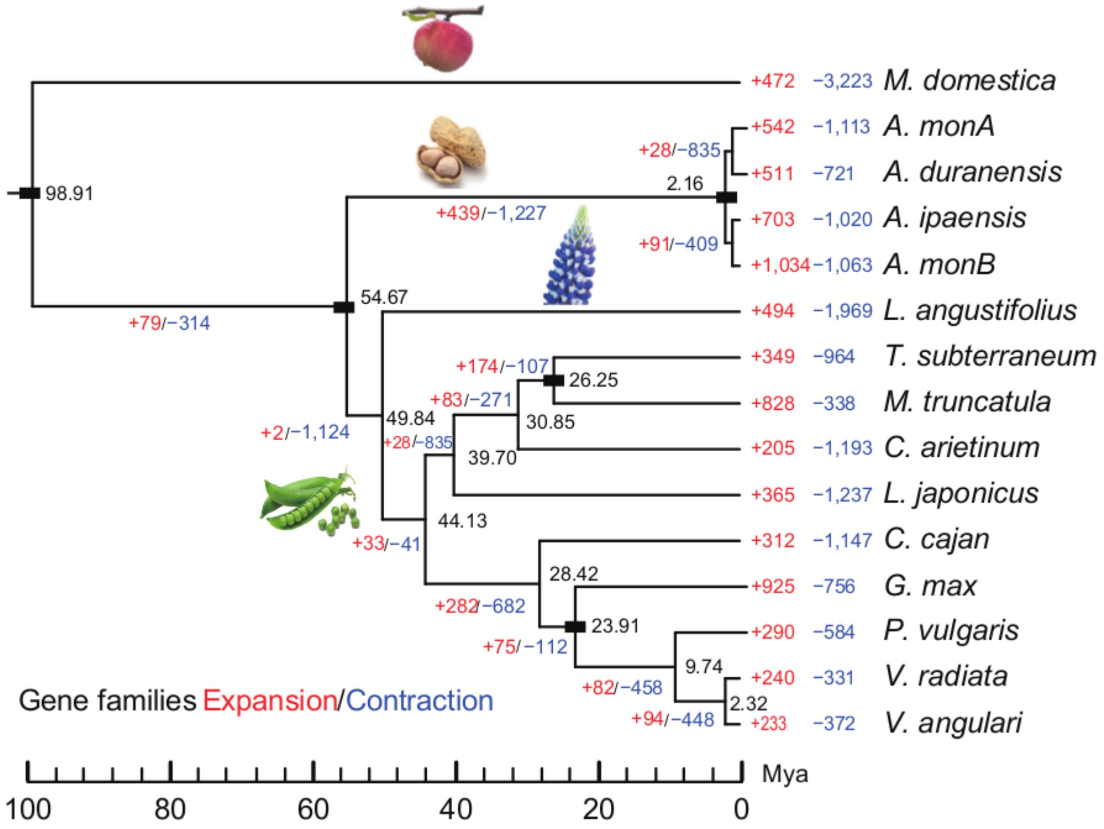

Supplementary Fig. 6 Phylogenetic relationship and gene family evolution of legume species with available genome sequences. The *M. domestica* was considered as outgroup. The nodes which marked with black rectangle indicated the calibration times obtained from TimeTree database (http://www.timetree.org/). Mya represents abbreviation of million year ago.

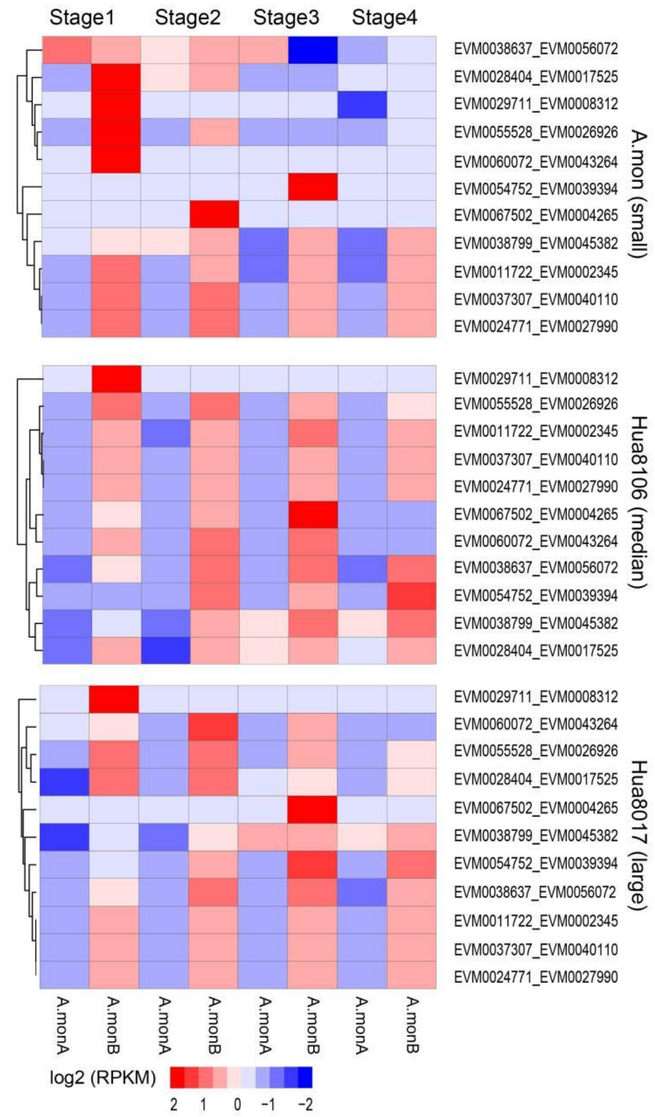

Supplementary Fig. 7 Expression bias of flavonoid biosynthesis related homeologous genes during pod development. The top, middle and bottom panels represent expression heatmap of wild peanut of *A. monticola* (small pod size), cultivated peanut of Hua8106 (median pod size) and cultivated peanut of Hua8107 (large pod size), respectively. The dominance behavior of flavonoid biosynthesis related genes is relatively conservative during different pod development stages and peanut lines, suggesting biological regulator in flavonoid biosynthesis.

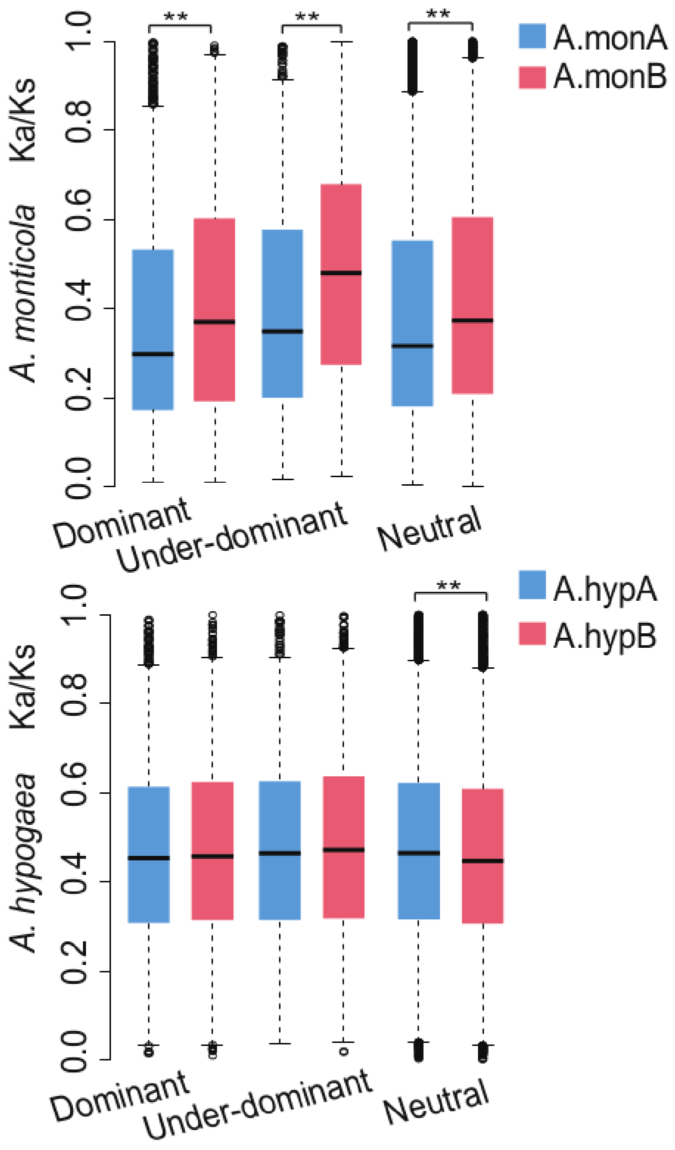

Supplementary Fig. 8 Selection bias among homoeolog expression dominance genes as dominant, under-dominant and neutral. The estimates were based on Ka/Ks values (Wilcoxon rank-sum test, ** indicates *P* value < 0.01). Upper panel represents the wild peanut *A. monticola*. Lower panel represents the cultivated peanut *A. hypogaea*.

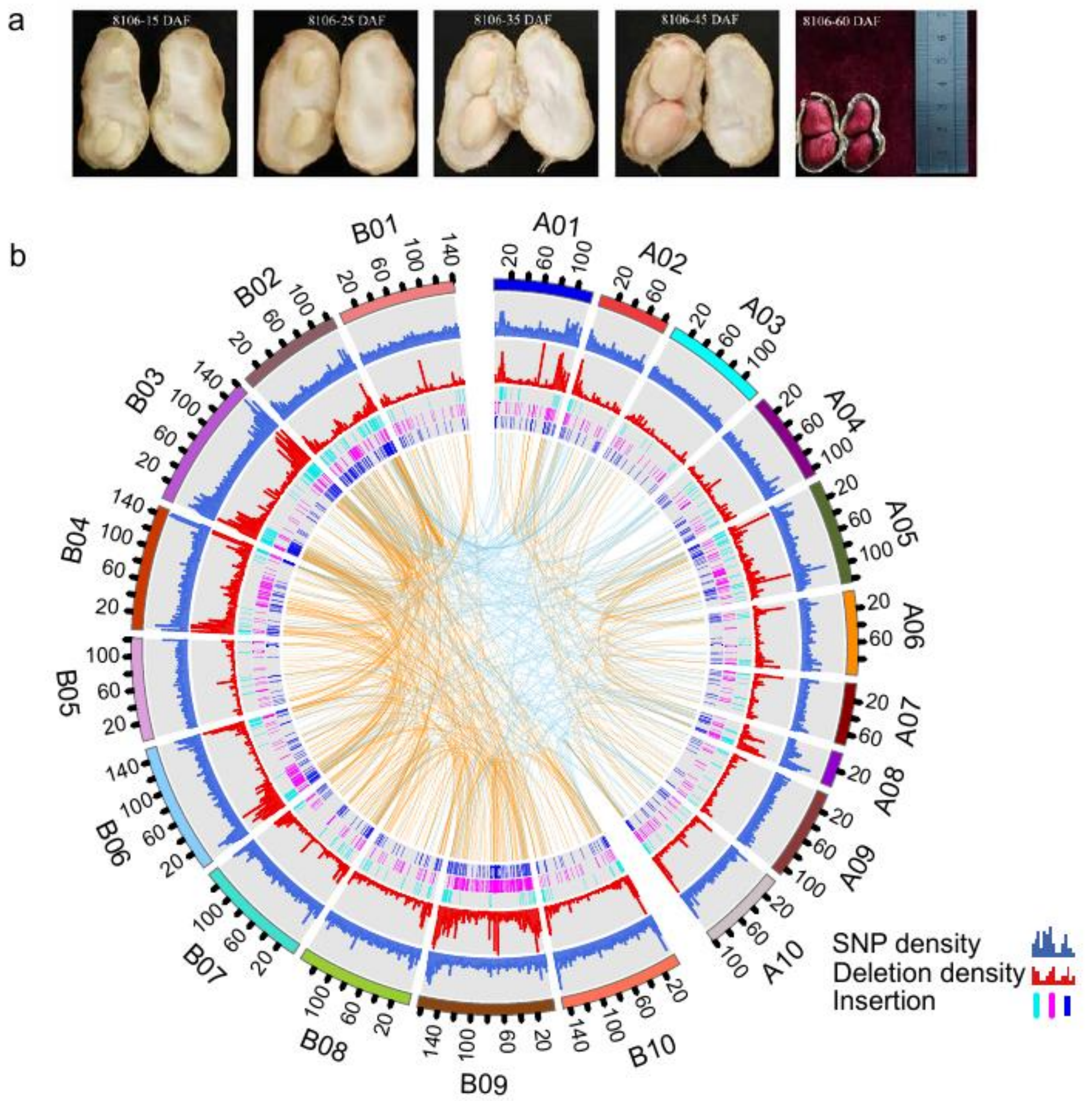

Supplementary Fig. 9 Photos of developing pod and genome-wide SV distribution for median pod lines. (**a**) Photos of developing pod from 15 DAF to 60 DAF for *A. hypogaea* (Hua8106: median pod). (**b**) Asymmetric SV abundance within subgenomes of *A. hypogaea* (Hua8106: median pod)

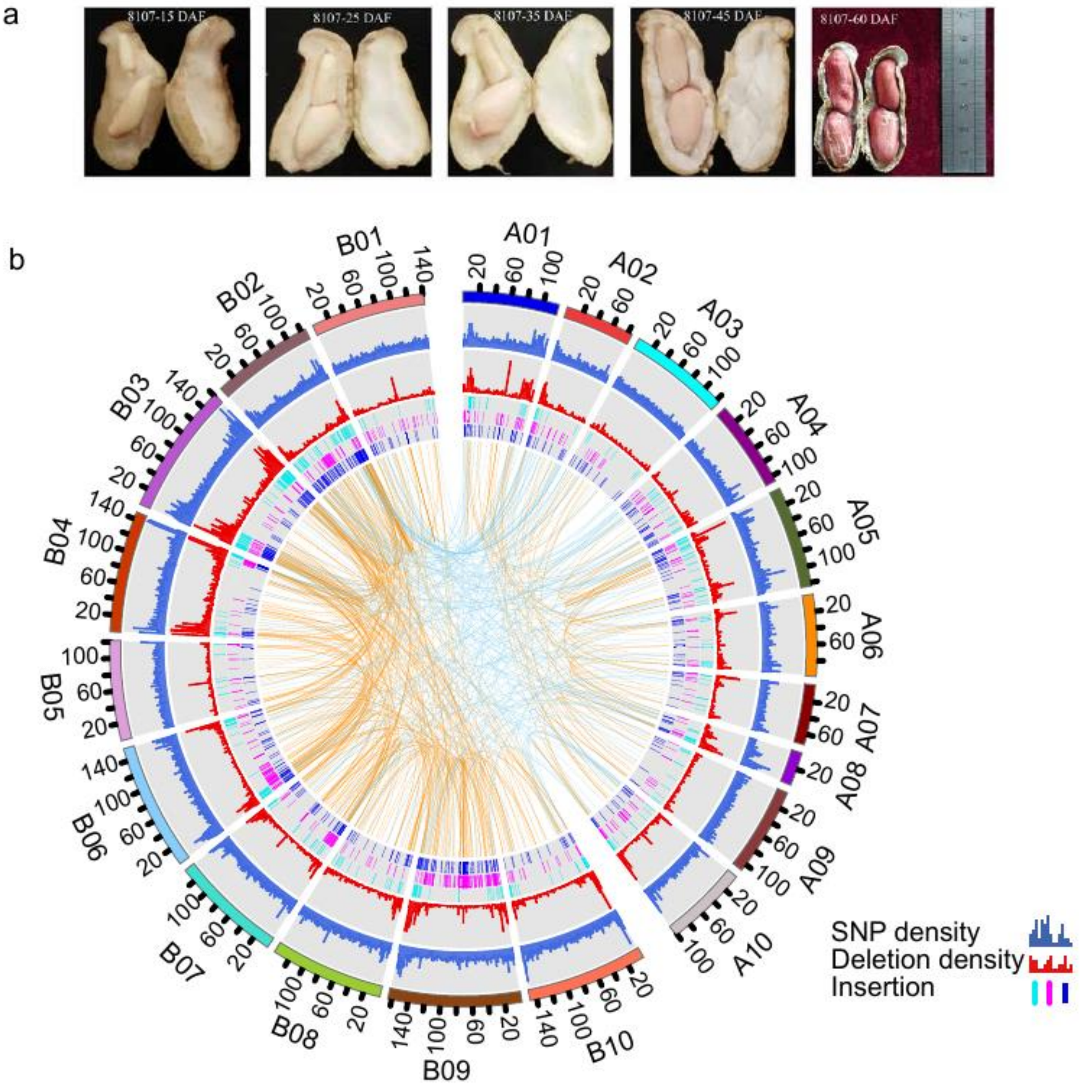

Supplementary Fig. 10 Photos of developing pod and genome-wide SV distribution for big pod lines (**a**) Photos of developing pod from 15 DAF to 60 DAF for *A. monticola* (Hua8107: large pod). (**b**) Asymmetric subgenome SV abundance within big pod cultivar accession (Hua8107: large pod)

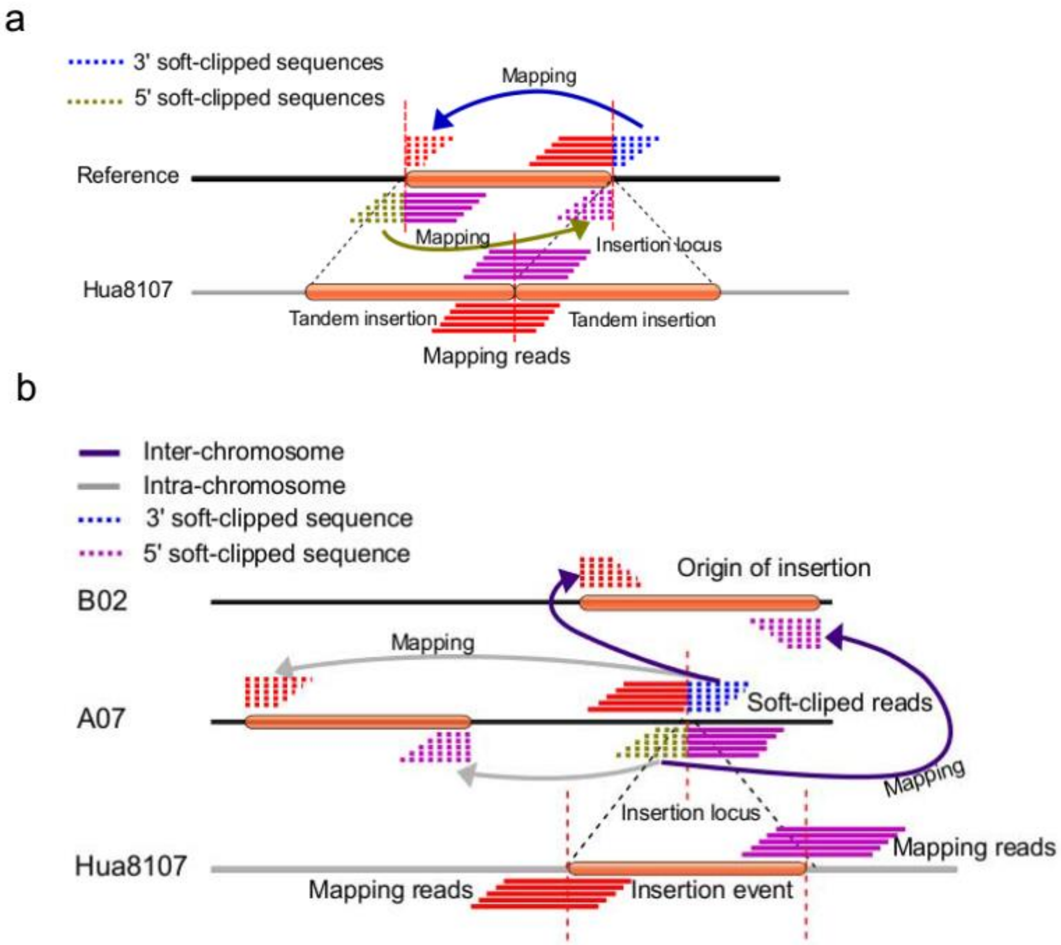

Supplementary Fig. 11 Illustration of definition for tandem insertion and confident origin insertion. (**a**) Illustration of identification pipeline for tandem insertion. (**b**) Illustration of identification pipeline for confident origin insertion.

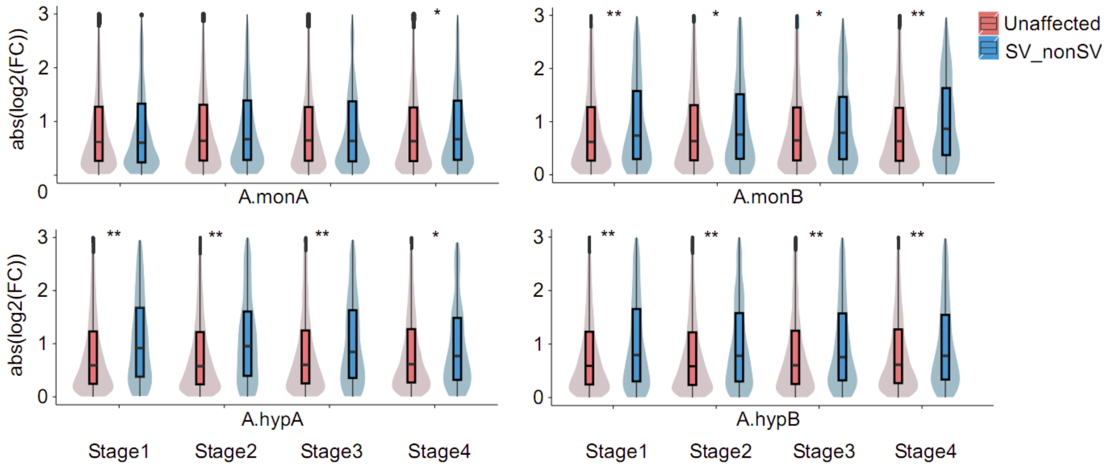

Supplementary Fig. 12 SVs were involved in expression divergence between homoeologous genes during different stages of pod development (Wilcoxon rank-sum test, *P < 0.05 and **P < 0.01).

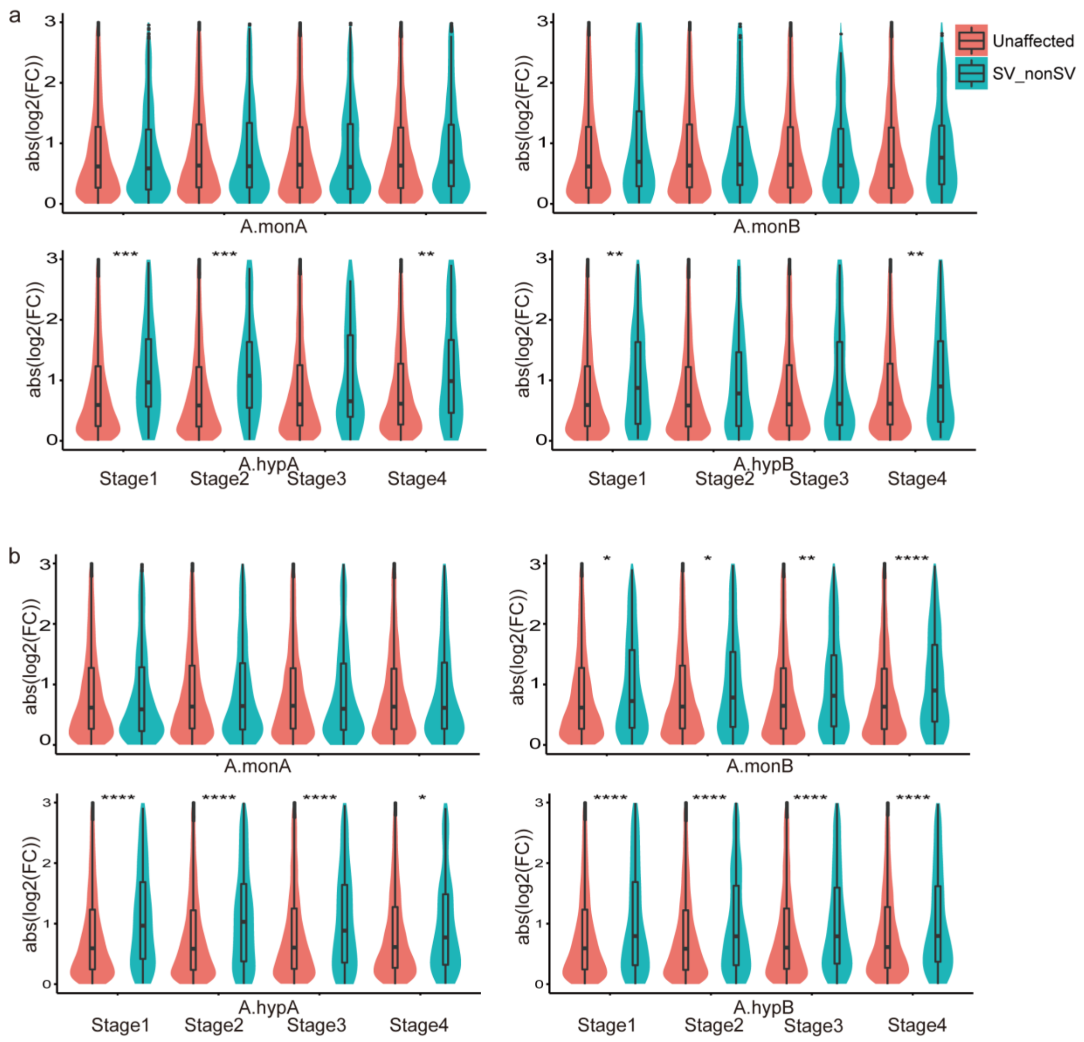

Supplementary Fig. 13 Insertion (a) and deletion (b) were involved in expression divergence between homoeologous genes during different stages of pod development (Wilcoxon rank-sum test, **P* < 0.05 and ***P* < 0.01).

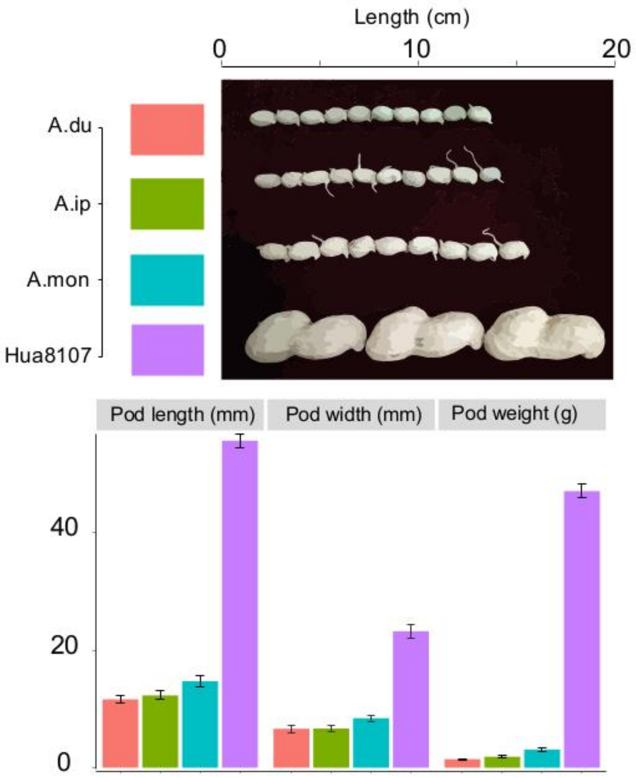

Supplementary Fig. 14 Investigation of length, width, and weight of pod among two ancestors (*A. duranensis* and *A. ipaensis*), wild (*A. monticola*) and cultivated (Hua8107) lines.

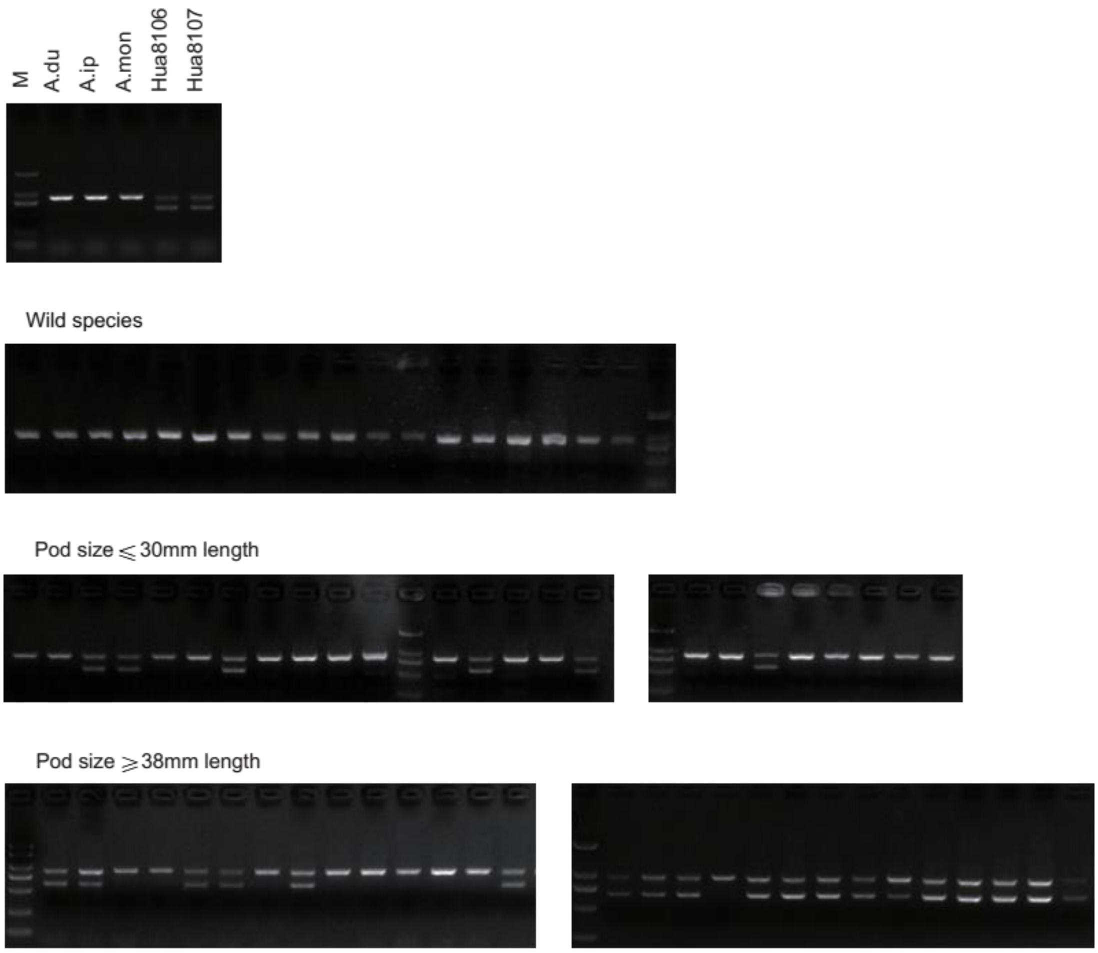

Supplementary Fig. 15 PCR validation of deletion in *ARF2-A08* in small pod, median and large pod lines.

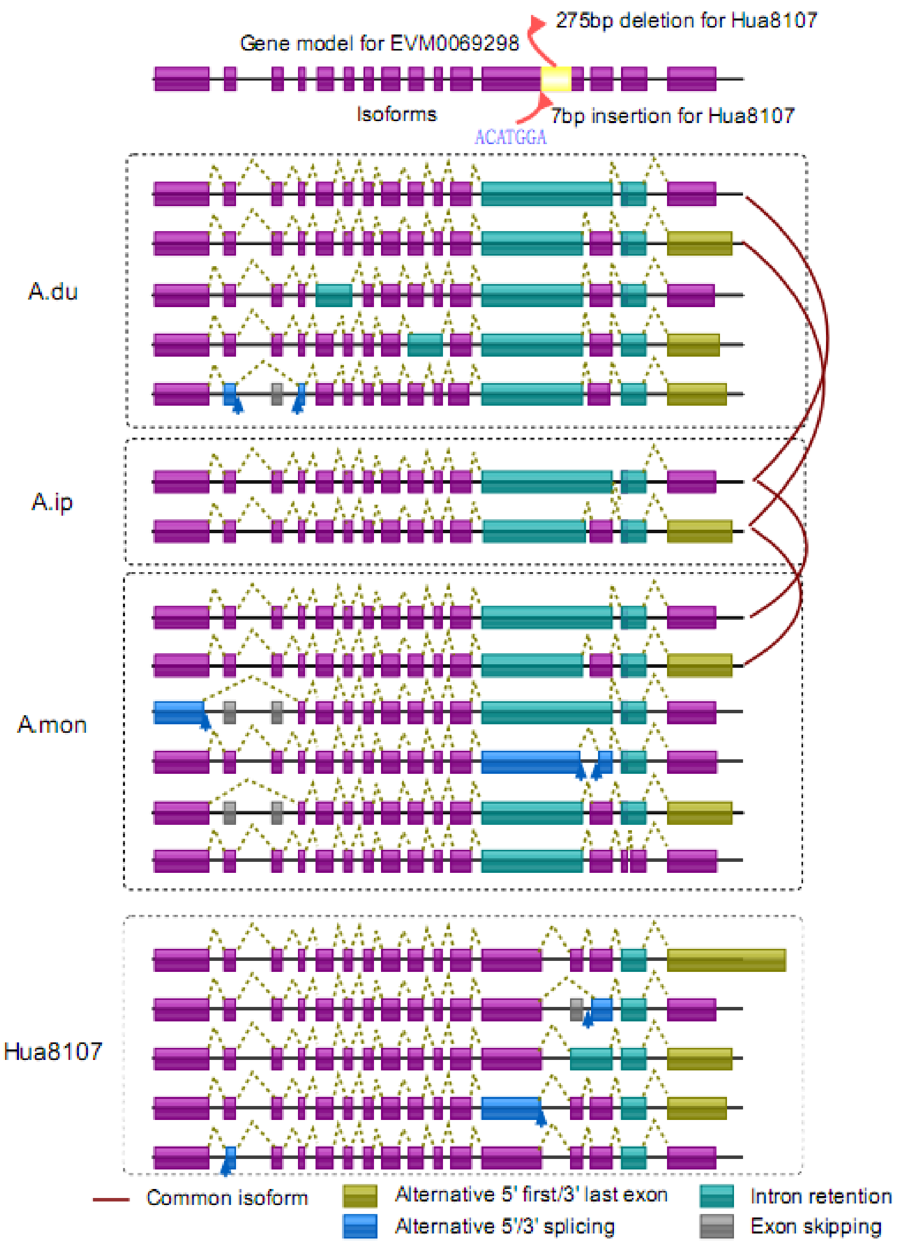

Supplementary Fig. 16 RNA splice models of *ARF2-A08* (*EVM0069298*) among wild and cultivated peanuts. Differently alternative sites occurred in the 12th exon, which has deletion mutation. The isoforms were supported by SMRT full length transcriptome sequencing data.

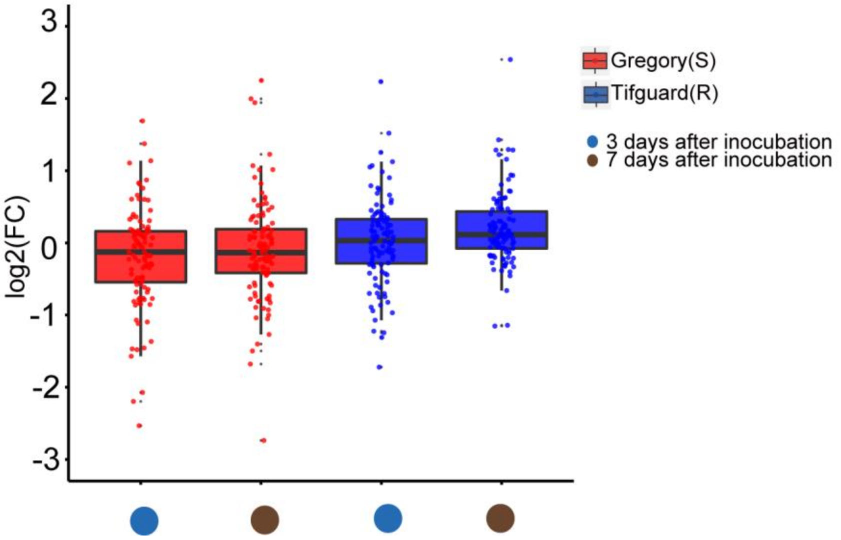

Supplementary Fig. 17 Expression pattern of SV-RGAs in root-knot nematode infection roots. The fold change value is calculated by RPKM(infected)/RPKM(control). The blue and red panels represent root-knot nematode susceptible and resistance groups, respectively.

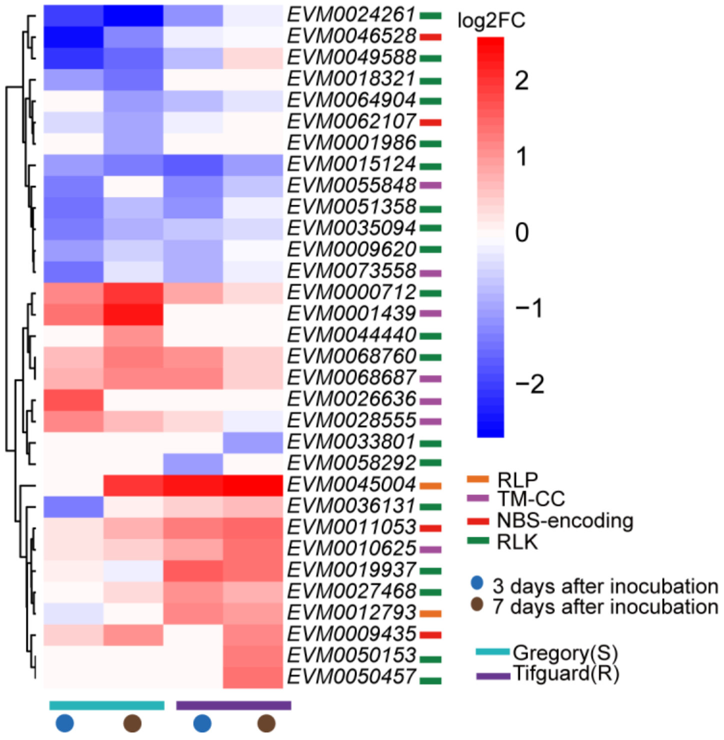

Supplementary Fig. 18 SV-RGAs (R genes with SV in genebody or upstream region) in response to root-knot nematode infection. SV-RGAs were defined as R genes

having SVs in genebody or upstream regions. SV-RGAs that exhibited differential expression (fold change >=2 and FDR <=0.05) after root-knot nematode infection were shown in the figure. Labels of Gregory(S) and Tifguard(R) represent susceptible and resistance cultivated peanut lines, respectively. CN, CC-NBS; CNL, CC-NBS-LRR; NL, NBS-LRR; RLK, receptor-like protein kinase; RLP, receptor-like protein; TMCC, transmembrane coiled-coil protein; TN, TIR-NBS; TX, TIR-unknown.
